# Late-stage changes in the composition of cell walls of maize plants expressing an apoplast targeted, senescence enhanced fungal ferulic acid esterase, and the subsequent effects on tissue saccharification

**DOI:** 10.1371/journal.pone.0315950

**Published:** 2025-01-03

**Authors:** Marcia M. de O. Buanafina, Phillip Morris, Sue Dalton, M. Fernanda Buanafina, Yijuan Wang

**Affiliations:** 1 Department of Biology, The Pennsylvania State University, University Park, PA, United States of America; 2 Institute of Grassland and Environmental Research, Plas Gogerddan, Aberystwyth, United Kingdom; University of Sao Paulo, BRAZIL

## Abstract

Using maize plants expressing an apoplast targeted *Aspergillus niger* ferulic acid esterase (FAEA), with FAEA driven by a *Lolium multiflorum* senescence enhanced promoter (*Lm*See1), we extended measurements of FAEA activity to late-stage senescing plants and measured the stability of FAEA activity following stover storage. The impact of FAEA expression on cell wall hydroxycinnamic acid levels and arabinoxylan (AX) cross-links, and on the levels of cell wall sugars, acetyl bromide lignin and sugar release following saccharification by a cocktail of cellulases and xylanases, was assessed during plant development to full leaf senescence. These were determined in both individual internodes and in combined leaves and combined internodes of FAEA expressing and control partner plants. FAEA expression was found to increase with plant growth up to the reproductive stage (R) of development in both stems and leaves but decreased as the leaves entered full senescence at R+ (18–20 d after R) stage. Moreover, FAEA activity was shown to be relatively stable over a six-month period following stover storage at 4°C. This FAEA expression resulted in significantly reduced levels of cell wall ferulates and diferulates in internodes. The internodes of late stage and senescing FAEA-expressing plants exhibited significantly improved saccharification with a cocktail of cellulase and xylanase enzymes at both the R and R+ stages of development.

## Introduction

Grass cell walls constitute a sustainable resource for several applications such as animal nutrition, biomaterial, and biofuel production. Bioconversion to fermentable sugars for ethanol production, provides a substitute for fossil-based, liquid transportation fuels, offering the potential to replace a significant fraction of petroleum-based fuels [1]. Food crops, such as corn, provide grain for humans, but the residue remaining after harvesting the stalks, leaves, husks, and cob is quite voluminous, with global annual estimates of one billion tons [2]. Due to its high carbohydrate content corn stover constitutes one of the most sustainable lignocellulosic biomasses for bioconversion to ethanol [3]. The use of grass cell walls for this purpose, however, requires the disassembly of the recalcitrant cell wall structure. This recalcitrance of plant biomass to degradation is a function not only of how cell wall polymers are deposited, but quite importantly, of the nature and extent of the cross-linking between polysaccharides.

Plant lignocellulosic biomass consists mainly of cell walls, a quite sophisticated component, which are composed of a combination of polymeric sugars (cellulose and hemicellulose) and phenolic compounds (lignin and hydroxycinnamic acids). One interesting aspect of corn and other grass species is that the hemicelluloses are largely arabinoxylans (AXs) (~30%) decorated with elevated levels of hydroxycinnamic acids (HCAs) (0.5–15% dry weight), of which ferulic and *p*-coumaric acid are the most abundant [4, 5]. AXs are polysaccharides with a linear backbone of β-(1–4)-linked D-xylanopyronosyl residues substituted with α-L-arabinose at positions *O*-3 and *O*-2, as the major substituents [6]. Acylation of AX by ferulic acid (FA) takes place predominantly via an ester-linkage at the C5-hydroxyl group of arabinose residues [7]. Ferulates are also ester, ether- and C-C linked to lignin [8–11] and can form dimers and trimers [12] upon oxidative coupling, mediated by peroxidase/H_2_O_2_ [13]. This process provides the conditions for FA cross-linking AX chains and AX with lignin [10, 11, 14, 15] and is among the key factors that increase cell wall recalcitrance because cell wall polysaccharides are protected from hydrolytic enzymes [16, 17].

Various approaches have been developed to modify plant cell wall structure/composition with the aim to overcome biomass recalcitrance, including genetic manipulation of model grass species and biomass feedstocks [18–21]. However, one challenge of modifying cell wall composition and structure is maintaining plant fitness and total biomass.

We have previously engineered different grass species expressing an *Aspergillus niger* ferulic acid esterase (FAEA) driven by the constitutive rice actin promoter, containing different signal sequences at the 5’ and/or 3’ ends of the coding region to confer different targeting [22, 23]. It was shown that constitutive vacuole, Golgi and apoplast targeted expression of the *A*. *niger* FAEA in *Festuca arundinaceae*, all reduced the level of cell wall ferulates monomers and dimers, but all expressing lines had shorter and narrowed leaves and shorter roots in the T_1_ progeny [24]. Genetic engineering of maize plants constitutively expressing apoplast targeted FAEA, also altered the level of cell wall ferulate monomers and dimers, but plant stature was severely compromised varying from semi-dwarf to extreme dwarfs with a significant reduction in biomass [25]. Alternatively, when FAEA was placed under a senescence enhanced promoter targeted to the apoplast (TQ plants), FAEA activity was found to be low up to the VT stage of plant development but then increased significantly at the R stage as the plants began to senesce. There was a negligible effect on plant development or biomass yield, but these TQ plants had reduced levels of cell wall ferulates throughout the stover of mature plants [25], indicating that the level of cell wall feruloylation can be genetically modified without compromising plant growth and biomass.

The aim of this work was to determine the levels of FAEA activity at the latter stages of plant senescence and examine how the subsequent changes in cell wall chemistry affected tissue saccharification.

## Material and methods

### Vector construction and maize transformation

The vector construct *p*JQ5 used to transform maize carried a genomic clone of the *Aspergillus* niger *fae*A gene which was under the *Lolium multiflorum* senescence inducible promoter (*Lm*::f*ae*A), targeted to the apoplast as previously described [19, 23, 25].

Maize hybrid genotype Hi-II (A188 x B73) was transformed, following the protocol from Iowa State University, by co-bombardment with *p*UBA containing a maize ubiquitin promoter-bar chimeric gene [26]. The T_0_ TQ generated plants were screened by PCR to verify the presence of the *fae*A gene and for FAEA enzymatic activity. T_1_ TQ plants were generated by back crossing FAEA and PCR positive plants to maize B73 or to negative FAEA plants.

### Plant growth conditions, plant material and tissue sampling

T_1_ transgenic and partner control wild type plants were grown in a glasshouse at Pennsylvania State University under a 16h/8h light/dark cycle at 29°C Day/ 27°C night, with supplementary 1000W metal halide lamps and fertilized weekly. Transgenic and control plants were analysed at the V6, V9, V15, VT and R and R+ developmental stages, according to the Iowa State University Manual [27], see also Buanafina et al. [22]. Plants at the R stage were harvested at 107–112 days after planting and at the R+ stage 127–130 days after planting.

A total of fifteen TQ plants expressing senescence inducible apoplastic targeted FAEA and thirteen control partner plants (C) were used in this study. Of these nine TQ plants (TQ4, 6, 10, 11, 12, 13, 17, 20 and 21), were used to determine FAEA activities in individual internodes and in pooled leaves at the various stages of plant development. Two plants (TQ20 and TQ21) were used to determine FAEA activities in different vegetative tissues and six plants (TQ10 & TQ13 at the VT stage), TQ12, TQ17 (at the R stage) and TQ20, TQ21 (at the R+), to determine ester-linked cell wall HCAs and sugar release following cellulase digestion. Two plants, (TQ20 and TQ21) were used to determine cell wall sugars, and four plants (TQ12, TQ17 at the R stage and TQ20, TQ21 at the R+ stage), to determine acetyl bromide lignin in individual internodes. Plant TQ22 was used to determine FAEA stability, and the levels of ester-linked HCAs. With plants TQ23-27 all the internodes from each plant were combined for analysis of ester-linked cell wall HCAs, cell wall sugars, acetyl bromide lignin and for tissue saccharification. The TQ plants were heterozygous T_1_ progeny derived from four independent T_0_ transformation events (a-d) backcrossed to B73 or to FAEA negative plants. Plants TQ12, TQ17 and TQ4 were derived from transformation event “a”; TQ10 and TQ13 from event “b”; TQ6 and TQ11 from event “c” and 4TQ20, TQ21, TQ22, TQ23, TQ24, TQ25, TQ26 and TQ27 from event “d”. Because the TQ plants were grown in several batches, they were allocated partner control plants that were grown and harvested in parallel with the transgenic lines. These control partner plants were T_1_ plants derived from FAEA negative plants backcrossed to B73.

### Stability of FEA activity on storage of stover

Plants harvested at the R stage were used to measure FAEA stability and the levels of ester-linked HCAs during a 24-week storage period. Internodes were harvested, chopped into small pieces and representative sub samples were taken to measure fresh weight and FAEA activity, then oven dried at 50°C to constant weight and used to determine tissue dry weight. The remaining internodes were split into two portions, and stored inside plastic containers, half at room temperature and the other half at 4°C. Three replicate samples were harvested at different time points during storage and assayed for FAEA activity and cell wall HCAs quantification.

Generally, for FAEA activity determination, whole longitudinal sections were taken from individual internodes to account for cell wall compositional differences among them. Analysis of leaves was carried out on pooled whole leaf blades where midribs were removed, leaves chopped, mixed, and sampled. All the remaining leaf and internode material was used to determine fresh and dry weights, ester, and ester+ether linked cell wall HCAs, cell wall sugars, acetyl bromide lignin and for saccharification experiments. For all other measurements sampled tissues were stored at -80°C until used for further analysis.

### Determination of FAEA activity

Enzyme activities were determined using ethyl ferulate as substrate as previously described [24, 25]. Briefly, soluble proteins were extracted with 0.1 M sodium acetate, pH 5.5 buffer containing 200 mg l^−1^ sodium azide, from pooled leaf blades and internodes, and 100 μg total protein was incubated with 24mM ethyl ferulate for 24h at 28°C, and FAEA activities determined by high-performance liquid chromatography (HPLC). One Unit of FAEA activity was defined as the release of 1μg ferulic acid from ethyl ferulate in 24h at 28°C. Therefore 1 Unit = 3.6 x10^-6^ μmole FA min^-1^.

## Cell wall analysis

### Preparation of isolated cell wall (AIR)

Representative samples of leaves and individual internodes harvested as described above, were ground in liquid nitrogen, freeze dried and milled to a fine powder using a mixer mill (Retsch MM301). For pooled internodes, the material was frozen to -80°C, freeze dried, and milled to a fine powder using a high-speed universal grinder (Cgoldenwall) before cell wall AIR was prepared as described in Zablackis et al. (1995) [28]; Fry (2000) [29] and Buanafina et al. (2015) [19] with modifications. Briefly, samples were shaken in 70% ethanol for 2 hours at room temperature, centrifuged and the residue washed (three times with 70% ethanol, twice with 1.5% SDS and 0.5% SDS, twice with 100% acetone, twice with 100% methanol and then three times with distilled water). The alcohol insoluble residue (AIR) of purified cell walls was then freeze-dried.

### Determination of cell wall ester and ether-linked hydroxycinnamic acids

The concentration of extracted ester and ester+ether linked HCAs was determined in isolated cell wall AIR samples by HPLC, as described in Buanafina et al. (2008) [22] with modifications. Briefly, esters were extracted with 1M NaOH at 25°C for 22h under N_2_, and ester+ether HCAs were extracted by treatment of AIR with 4M NaOH under N_2_ in a Teflon bomb (Parr Instrument Co) in a forced air oven at 170°C for 3 hours. The internal standards 2-hydroxycinnamic acid and 3-hydroxy-4-methoxycinnamic acid (100 μg) (Sigma) were added, for ester and total ester+ether linked HACs quantification respectively, to correct for losses during extraction. The extracted phenolics were loaded onto an activated reverse-phase C18 μNova Sep-Pak column (Waters Inc.) and eluted with 100% methanol. Samples were analysed by HPLC on a Nova-Pak C18 4 μm (3.9 × 75 mm) Radical-Pac-Cartridge (Waters Inc.) in 100% methanol–5% acetic acid, with either a 5%–80% methanol gradient over 15 min (FAEA assay) or a 10%–80% methanol gradient over 25 min (for monomeric and dimeric cell wall components) at a flow rate of 1 mL/min. Phenolic compounds were detected and quantified with a Waters 996 photo-diode array detector, with UV–visible spectra collected at 240–400 nm, and analysed using Millennium software (Waters Inc.) against authentic monomer standards, or using response factors for the various dehydrodiferulate dimers as in Waldron et al. (1996) [30].

The HCAs quantified by HPLC-PDA in the extracts were *p*-coumaric acid (*p*CA), ferulate monomers (FM) (consisting of cis and trans ferulic acid), ferulate dimers (FD) [consisting of 8–5’-DFA (or 8–5 benzofuran DFA); 5–5’-DFA; 8-O-4’-DFA; 8–5’-DFA (benzofuran cyclic form)] and an unknown FD quantified as for ferulic acid, as described previously [22].

### Determination of hydroxycinnamic acid conjugates released by mild acid hydrolysis

Mild acid hydrolysis was performed on combined internodes from JQ, and control plants as described in Saulnier et al. (1995) [31] and Dwivedi et al. (2023) [20] with modifications. In brief, AIR (10 mg) was hydrolysed in 1 ml of 50 mM TFA and incubated at 99°C for four hours under shaking at 700 rpm. After centrifugation at 10,000 g for 10 minutes, 400 μl of supernatant was freeze dried. The pellet was washed with acetone and water and freeze dried. The dried pellet and supernatant were incubated with 700 μl of 2M NaOH at RT for 16 hr and the level of HCAs was determined as described above. Mild acid hydrolysis was also performed on cellulase digests from control plants in order to determine the composition of HPLC peaks in the extracts treated with *T*. *reesei* cellulase or cellulase +GCI140 xylanase or CTec3 HS, not present in FAEA expressing plants. The internal standard 2-hydroxycinnamic acid (10 μg) (Sigma) was added, for ester linked HACs quantification, to correct for losses during extraction.

### Determination of cell wall sugars

To determine cell wall xylose, arabinose and glucose levels in individual internodes, cell wall AIR samples were hydrolysed in 1 ml of 2 M trifluoracetic acid (TFA) at 120°C for 1h, and TFA removed with a gentle stream of air flow at 25°C. The hydrolysate was then resuspended with double distilled water, diluted, and filtered through a 0.2 μm nylon filter and the filtrate was analysed by high-performance anion-exchange chromatography (HPAEC) with pulsed amperometry detection (HPAEC-PAD) on a Dionex ICS-300 system with the CarboPac PA20 column (3 x 150 mm), based on methods from Øbro et al. (2004) [32] with modification as described in Buanafina et al. (2012) [33].

For combined internode sugar analysis, two hydrolysis steps were carried out based on Saeman et al. (1963) [34]; Sluiter et al. (2008) [35] and Wilson et al. (2021) [36]. Cell wall AIR samples were first hydrolysed in 1 ml of 2 M TFA (as described above). The supernatant was then separated from the residue and evaporated with a gentle stream of air flow at 25°C. The dried supernatant was resuspended, diluted, and filtered as above for HPAEC. The residue was treated with 100 μl 72% sulphuric acid (SA) (second hydrolysis), shaken at 25°C until the samples were thoroughly dissolved and then 900 μl of ddH_2_O was added and samples heated at 120°C for 1 hr. Samples were allowed to cool on ice and then neutralized with 6M NaOH (to pH ~7, monitored with pH paper). Volumes were adjusted to 2ml, and samples filtered through a 0.2μm PVDF syringe filter for HPAEC. Results from HPAEC were corrected for glucose, arabinose and xylose using glucose, arabinose and xylose standards that were taken through the hydrolysis to correct for losses of sugars during acid hydrolysis.

### Biomass saccharification and sugar determination

#### Cellulase-mediated glucose release from internodes

For individual internodes, freeze-dried powdered internode material (30 ± 0.2 mg), was re-hydrated in 1.8 ml 0.1 M sodium acetate, pH 5.5 extraction buffer and incubated at room temperature for 24 h with shaking. After centrifugation, the supernatant was removed and the pellets washed twice with buffer and incubated with 160 μl cellulase (*T*. *reesei*, Sigma, 63 units/ml) and 4 μl 2% sodium azide in a total volume of 1.8 ml of the same buffer. Samples were incubated at 37⁰C for 48 h with shaking. Reactions were terminated by placing samples at 99°C for 5 minutes. The amount of sugar released after enzymatic saccharification was determined by the ρ-hydroxybenzoic acid hydrazide (PAHBAH) method [37] using glucose as standard as described in Buanafina et al. (2010) [23].

For combined internodes, cell wall AIR and unextracted material, 20 ± 0.2 mg dry weight, was incubated in 100mM sodium acetate, pH 5.5 buffer at 37°C with either *Trichoderma reesei*, cellulase (Millipore) and *T*. *reesei* endo-1,4-β-xylanase GC 140 (Genencor Inc.), or with a proprietary enzyme mixture (Cellic CTec3 HS, Novozymes), in 50mM sodium acetate pH 5.0 buffer at 45°C both containing 18 μl 2% sodium azide, in a final volume of 1.8 ml for up to 72 hours with shaking. The following four enzymatic treatments were used: **1.** 50–5000 U g^−1^ dry weight AIR Cellulase (*Trichoderma reesei*); **2.** 2,500 U g ^−1^ dry weight AIR Cellulase (*T*. *r*.) + 100–5000 U g ^−1^ dry weight AIR endo-1,4-β-xylanase (GC 140); **3.** 5,000 U g ^−1^ dry weight AIR Cellulase (*T*. *r*.) + 5,000 U g ^−1^ dry weight AIR endo-1,4-β-xylanase (GC 140) or **4.** 50–1000 U g ^−1^ dry weight AIR of CTec3 HS.

The amount of reducing sugar released after enzymatic saccharification was determined by the *p*-hydroxybenzoic acid hydrazide (PAHBAH) method [37] using glucose as standard, as described in Buanafina et al. (2010) [23]. The glucose, xylose and arabinose content after enzymatic saccharification was also measured by HPAEC-PAD as described in the previous section. Enzymatic saccharification efficiency was measured as the percentage of reducing sugars released from total sugars measured in non-digested cell wall AIR material.

Mild acid hydrolysis (as described in previous section) followed by saponification was also performed on 500 μl of the enzymatic digests of internode AIR from control plants, in order to test if chromatogram peaks contained feruloylated cell wall components.

### Determination of acetyl bromide lignin

The acetyl bromide lignin content of individual and combined internode material was analysed by the acetyl bromide (AB) method [38] as described in Buanafina et al. (2015) [19], using 5 ± 0.1 mg of extractive-free material (prepared according to Dean,1997) [39]. Acetyl bromide lignin concentrations were calculated using a molar extinction coefficient of 17.75 cm^2^ g ^−1^ derived from purified HCL-dioxane lignin isolated from corn stems [40].

### Statistical analysis

Statistical Analysis System (SAS) (2015) [41] software version JMP Pro 17 (SAS Institute Inc., Cary, NC) was used to perform all statistical analysis. Values in the text are means ± the standard error of the means (sem). Bars with asterisk are significantly different from controls (Student’s *t*-test α = 0.05).

## Results

### FAEA expression under the *Lolium multiflorum* senescence promoter increases at later stages of plant development

**Fig 1:**
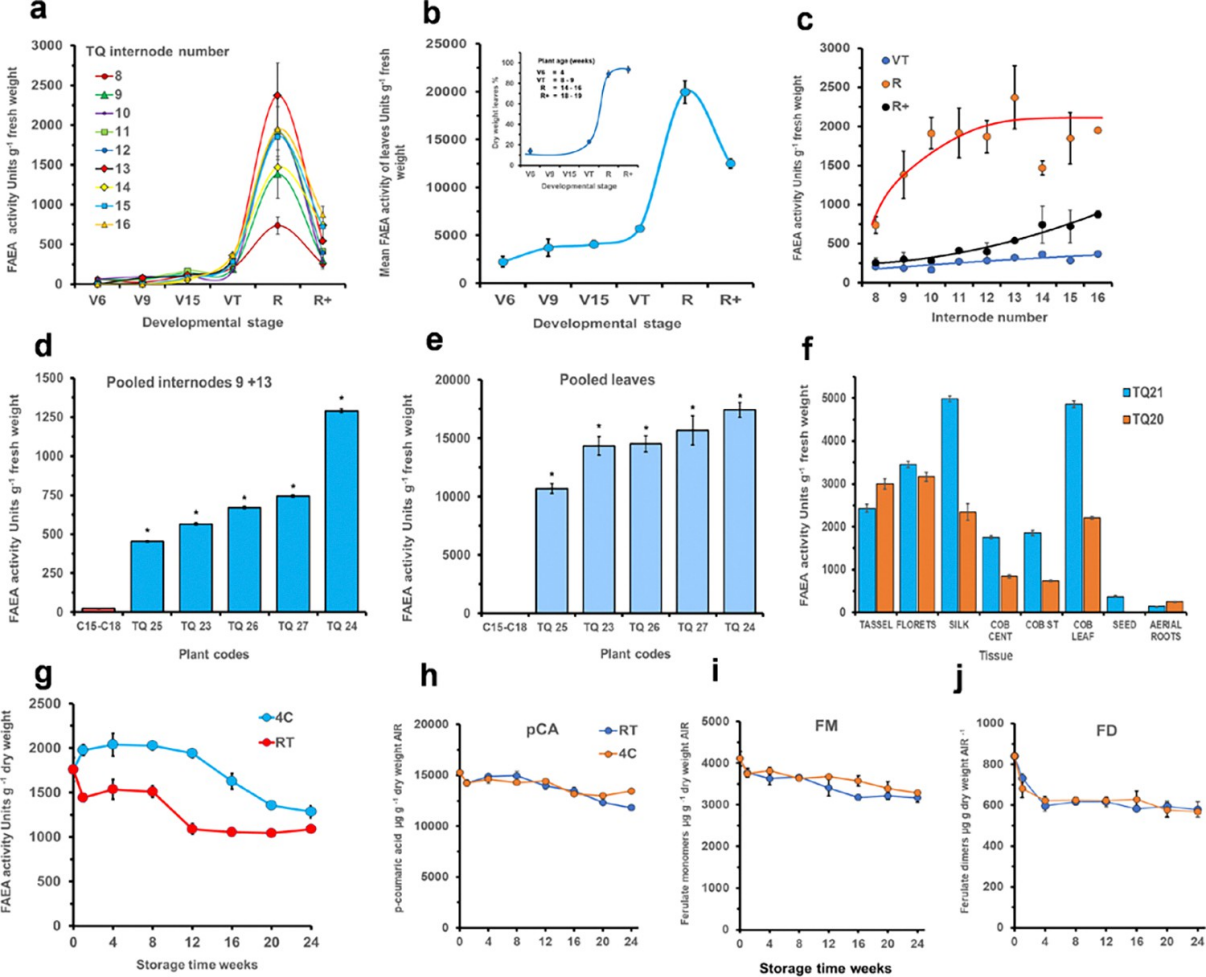
Accumulative maximum FAEA activity in internodes 8–16 from V6 to R+ developmental stages [plants TQ11 (V6), TQ4 (V9), TQ6 (V15), TQ10&TQ13 (VT), TQ12&TQ17 (R) and TQ20+TQ21 (R+)]. V6—VT are means ± sem (n = 3) from single plants. R and R+ are the means of two plants ± SD (n = 6)] **(a).** Maximum leaf FAEA activity on a fresh weight basis, with insert showing mean % dry weight of leaves at each stage **(b).** FAEA activity in individual internodes at VT, R and R+ **(c).** FAEA activity of internode 9+13 from 5 replicate TQ plants **(d)** and of pooled leaves **(e)** of 4 control plants and 5 TQ plants at the R stage of development [Mean ± sem (n = 3)]. FAEA activity at the R+ developmental stage (plantsTQ20 and TQ21) in different vegetative tissues (COB CENT = Cob centre, COB ST = Cob outer). Means ± sem (n = 2) **(f)**. Post-harvest stability of FAEA activity in pooled internodes of plant TQ22 at the R stage of development **(g)** and levels of ester-linked cell wall hydroxycinnamic acids **(h-j)** during subsequent storage either at room temperature (RT) or at 4°C (4C) for 24 weeks. Bars are mean ± sem (n = 3). *p*-coumaric acid (*p*CA). Ferulate monomers (FM), Ferulates dimers (FD). * Indicates significant differences from controls (Student’s α = 0.05).

When under the *Lm See1* senescence promoter, the level of FAEA activity in TQ internodes was found to be very low up to the VT stage of plant development but increasing significantly at the R stage in internodes 8–16 (Fig 1A), confirming that FAEA expression in internodes of TQ lines increases as senescence begins and that FAEA activity was not induced until after internodes had passed their period of rapid elongation [22]. In order to establish if FAEA activity increased further or declined on prolonged plant senescence after the R stage, FAEA expression was measured at the R+ stage in individual internodes (Fig 1C) and in fact, FAEA activities were found to decline after the R stage in both internodes and in leaves (Fig 1A and 1B).

Because FAEA activity was measured on individual internodes from early (V6) to senescing (R+) developmental stages, we were also able to show the maximum FAEA activity in internodes at each stage and to confirm the increase in dry weight as plants senesced (S1A Fig), as well as to determine which internodes contributed the highest FAEA activity at each developmental stage (S1B Fig). The maximum FAEA activity in pooled leaves at stages V6 to R+ was found to show a similar pattern of expression to internodes, with higher levels on a fresh basis at the R stage (Fig 1B). However, most of this increase in FAEA activity in leaves may be accounted for by the increase in % leaf dry weight as the leaves senesced and dried (Fig 1B insert) although FAEA activity was also found to increase on a dry weight basis between V6 and VT stages, but then declined at the R+ stage (S1C Fig).

Significant FAEA expression was also confirmed in selected maize internodes at the R stage, as shown for internodes 9+13 from five replicate TQ plants (Fig 1D) with activity levels varying between different plants. In pooled leaves, FAEA activities were much higher compared with internodes and other vegetative tissues (Fig 1E) and levels also varied between plants. FAEA expression was also evident in other vegetative maize tissues when examined at the R+ stage. FAEA activities intermediate between internodes and leaves were found in tassels, florets, silks, and in both the centre and outer parts of the cob, albeit at variable levels (Fig 1F).

### Stability of FAEA during post-harvest storage of stover

Whether FAEA expression driven by a senescence promoter continued post-harvest, the stability of FAEA activity during the storage of the stover, and whether there were further reductions in cell wall HCAs were investigated by storing internodes at 4°C and at room temperature for 6 months. When stored at 4°C, there was no decrease in FAEA activity within the first 12 weeks and only a 22–27% decrease after 24 weeks stover storage. Storage at room temperature led to some loss in FAEA activity ranging from 12–18% within the first 8 weeks, and up to 38–40% loss after 12 weeks (Fig 1G).

Quantification of cell wall HCAs in stover during the storage period showed that there was a small but steady decline in the level of both monomeric and dimeric ester linked cell wall ferulates (Fig 1H–1J), when stored at room temperature or at 4°C.

### Chemical phenotypes of FAEA expressing plants

#### Late-stage reductions in the level of ester-linked HCAs in internodes of TQ plants

**Fig 2:**
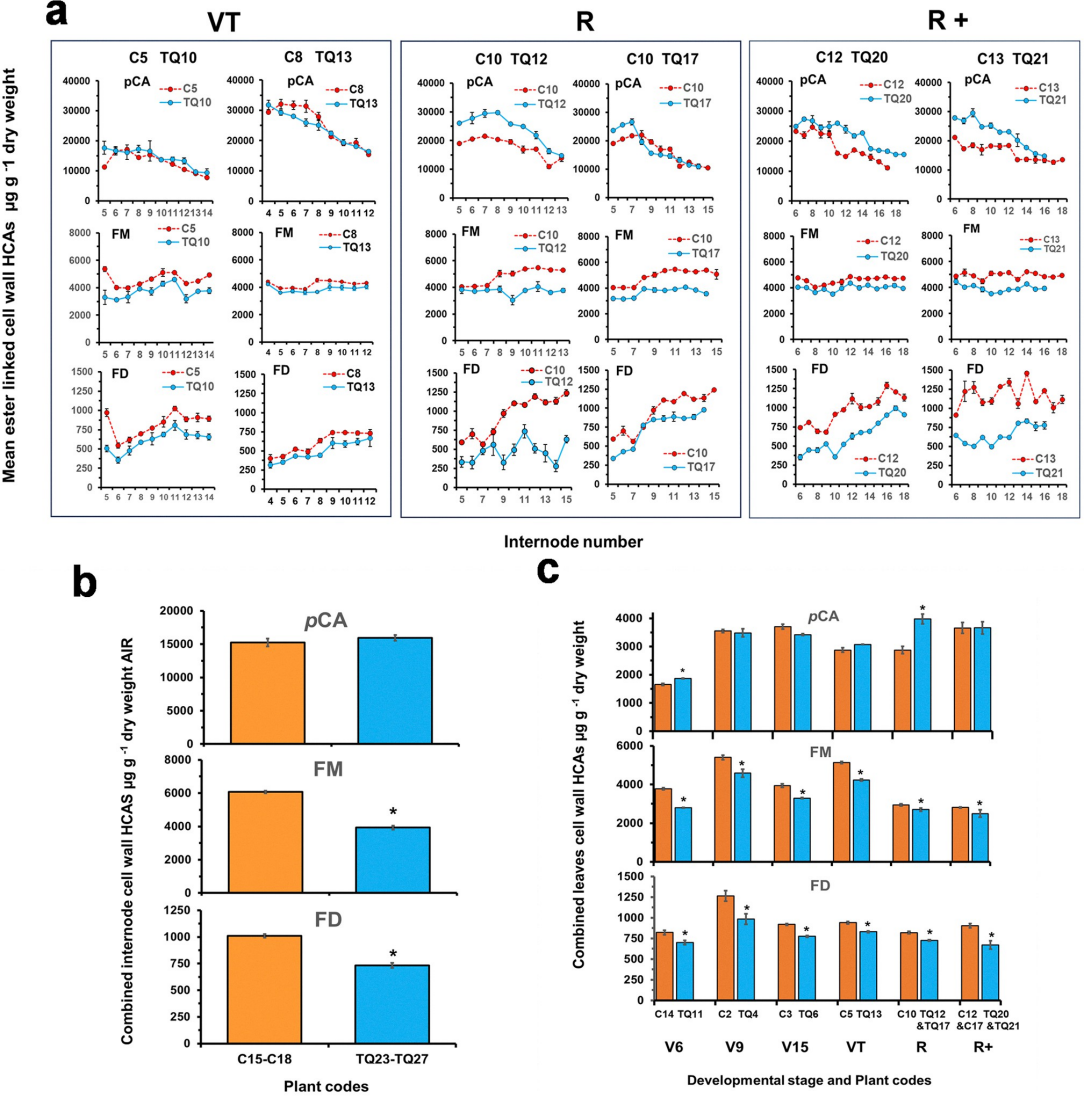
Levels of ester linked cell wall hydroxycinnamic acids in individual internodes of replicate FAEA expressing and partner control plants at the VT (TQ10 & TQ13), R (TQ12 & TQ17) and R+ (TQ20 & TQ21) developmental stages. *p*-coumaric acid (*p*CA). Ferulate monomers (FM), Ferulate dimers (FD). Error bars are mean values ± sem (n = 2–3) **(a)**. Mean levels of ester-linked HCAs in the cell walls of combined internodes from single plants of 5 FAEA expressing TQ plants (TQ23-TQ27) and 4 partner control plants (C15-C18) at the R stage of development. Error bars are mean values ± sem (n = 13–20) **(b)**. Mean levels of ester-linked HCAs in pooled leaves of TQ and C plants at V6 to R+ stages of development. Error bars are mean values ± sem (n = 3) **(c)**. * Indicates significant differences from controls (Student’s α = 0.05) (P<0.0001).

The ester-linked HCAs, *p*-coumaric acid, ferulate monomers and ferulate dimers in individual internodes of replicate TQ and control plants were determined at the VT, R and R+ stages of development (Fig 2A).

At the VT stage of development, FAEA expression had little effect on *p*-coumaric acid as the levels were similar between the transgenic plants TQ10 and TQ13 and respective wild type partner control plants in most internodes (Fig 2A). In contrast, there was a modest reduction in the level of ester-linked ferulate monomers in internodes 4–14 of TQ10 and TQ13 when compared to respective control plants, with the earlier formed internodes showing smaller reductions than the latter ones. Similar effects were observed for ester-linked ferulate dimers in internodes of TQ10 and in TQ13, when compared with the corresponding controls (Fig 2A) which is consistent with FAEA expression in the internodes of FAEA expressing plants at the VT stage (Fig 1A and 1C). At both the R and R+ stages, FAEA expression in the individual internodes resulted in a larger reduction of ester-linked ferulate monomers and dimers, but a 6–48% increase in *p*-coumaric acid compared with controls (Fig 2A).

In latter experiments to determine saccharification efficiency, a different set of TQ plants was generated and harvested at the R stage of development. The effect of FAEA expression on cell wall HCAs, and cell wall sugars was therefore verified using samples from pooled internodes of five TQ plants and corresponding controls (Fig 2B). The effect of FAEA expression on the level of ester-linked HCAs, measured in pooled internodes of these new transgenic plants at the R stage, showed that, there was a significant increase in the level of *p*-coumaric acid in two out of five of TQ plants (S7B Fig) and a significant reduction in the level of ferulate monomers and dimers (Fig 2B, S7B Fig) among FAEA expressing plants compared with controls. Pairwise comparisons between pooled internodes of TQ plants and controls are shown in S7B Fig.

### Levels of ester-linked HCAs in leaves of TQ plants are reduced during plant development

The high levels of FAEA activity found in pooled leaves in TQ plants from early developmental stages (V6) through to senescence (R+) (Fig 1B) also resulted in significantly lower levels of ester-linked monomeric and dimeric ferulates in the leaves than in controls for all developmental stages studied (Fig 2C). Also, as with internode (Fig 2A), levels of ester-linked *p*CA significantly increased in leaves at the R stage (Fig 2C). Pairwise comparisons between TQ plants and respective control plants showed a statistically significant increase in the levels of *p*CA for TQ11 (V6 stage) (P< 0.0493) and TQ12+TQ17(R stage) (P < 0.0013) and significantly decreased levels of ferulate monomers and dimers for all TQ plants (P< 0.0001).

### Levels of total (ester+ether) linked HCAs are reduced in selected internodes of plants at the VT stage of development

In internodes (9–12) of plants TQ13 and TQ10 at the VT stage of development, the total ester and ether-linked monomeric and dimeric ferulates were consistently lower compared with control (S3A–S3D Fig) The *p*-coumaric acid levels were in general also lower in internodes of TQ10 and TQ13 plants compared with controls but only statistically significant different for internode 11 (P<0.0017) (S3A–S3D Fig).

### Reduced levels of hydroxycinnamic acid conjugates also confirmed by mild acid hydrolysis

In order to get further information about which cell wall fraction showed altered levels of FA and *p*CA in FAEA expressing plants, the AIR from internodes of two TQ plants and a control were subjected to a mild 50 mM TFA hydrolysis. This treatment cleaves arabinofuranose [arabinosyl-(1–3)-xylan] glycosidic linkages within glucouroarabinoxylan chains leaving esters of ARA reasonably, although not completely intact, enabling quantification of AX-bound HCAs and ferulates coupled to lignin (remaining in the residue) [31, 42]. TFA supernatants and residues were then subjected to saponification and the products analysed by HPLC. The results confirmed ferulic acid association with the TFA fraction with less than 10% ferulic acid associated with the lignin in the residue, in both TQ and control plants and also revealed a significant reduction of ferulic acid associated with both arabinoxylan (TFA fraction) or with lignin (residue) in TQ plants (S2D Fig). Pairwise comparisons between TQ23 and TQ25 and control plants confirmed statistically significant reduced levels of ferulic acid ester-linked to ARA (P< 0.0045) and to lignin (residue) (P< 0.001) in TQ plants (S2D Fig). By contrast, around 90% of *p*CA remained ester-linked to lignin (residue) and less than 10% remained in the TFA fraction (S2E Fig).

### Late-stage changes in acetyl bromide lignin in internodes of TQ plants

**Fig 3:**
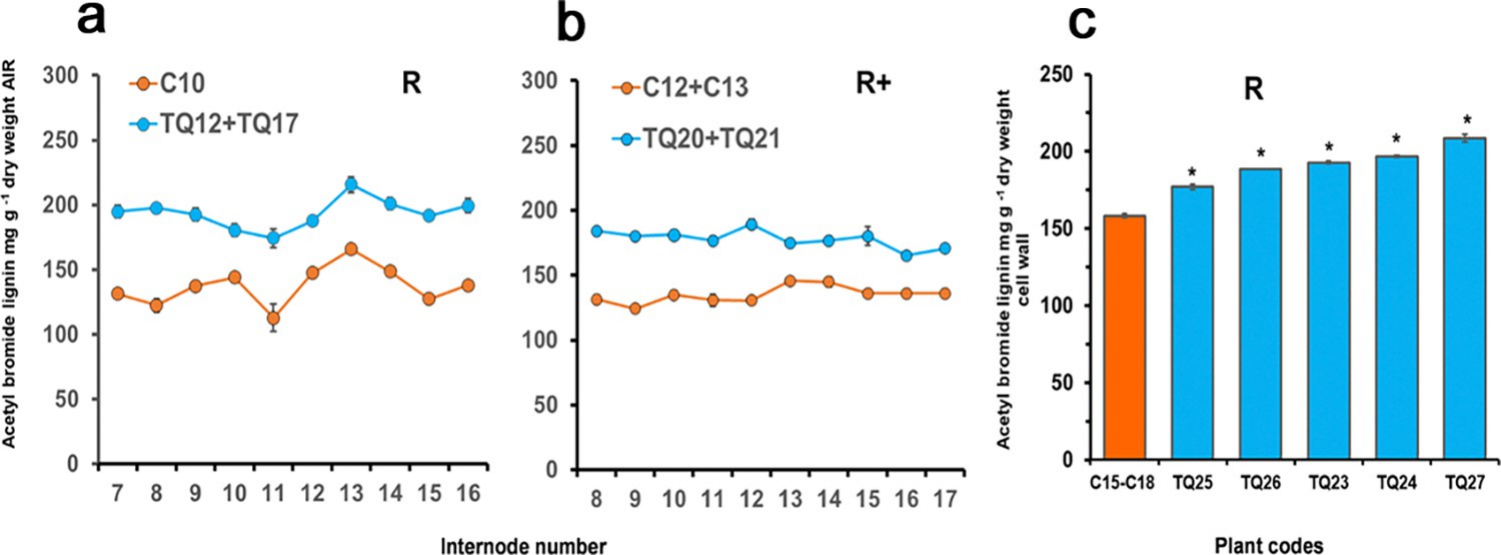
Mean levels of acetyl bromide lignin in individual internodes of two FAEA expressing and partner control plants at R **(a)** and R+ **(b)** developmental stages. Means ± sem [(n = 4–12 **(a)** & 12–18 **(b)**]. Mean levels of acetyl bromide lignin of combined internodes of four control (C15-C18) and five independent TQ plants (C23-C27) at the R stage of development **(c, d)**. Error bars are mean ± sem (C n = 18, TQ n = 24). * Indicates significant differences from controls (Student’s α = 0.05). (P<0.0.0001–0.0012).

As FAEA expression has the potential to disrupt ferulates esterified dimer linkages to arabinoxylans and lignin and hence arabinoxylan-ferulate-lignin linkages, we hypothesized that FAEA expression would increase lignin solubility and as such, the AB method was used to test this hypothesis. The AB method is an indirect method for lignin quantification that involves the acetylation of lignin unsubstituted γ-hydroxy groups and bromide substitution of its α-hydroxy groups and its solubilization in acetic acid. The solubilized lignin is quantified using spectrophotometer at 280nm [43]. Results showed that the AB values for all the individual internodes of plants TQ12+TQ17 at the R stage (Fig 3A), and TQ20+TQ21 at the R+ stage (Fig 3B) and all 5 of the combined internode FAEA expressing plants at the R stage (Fig 3C and 3D), were higher when compared with control plants. Pairwise comparisons between internodes from transgenic and controls showed statistically significant increased values for acetyl bromide for all individual internodes.

### Late-stage specific changes in the composition of cell wall sugars in internodes of TQ plants at the R and R+ stage of development

The effect of FAEA expression on the composition of cell wall arabinoxylan and glucose of both individual internodes at the R+ stage and of combined internodes at the R stage of development was determined.

**Fig 4:**
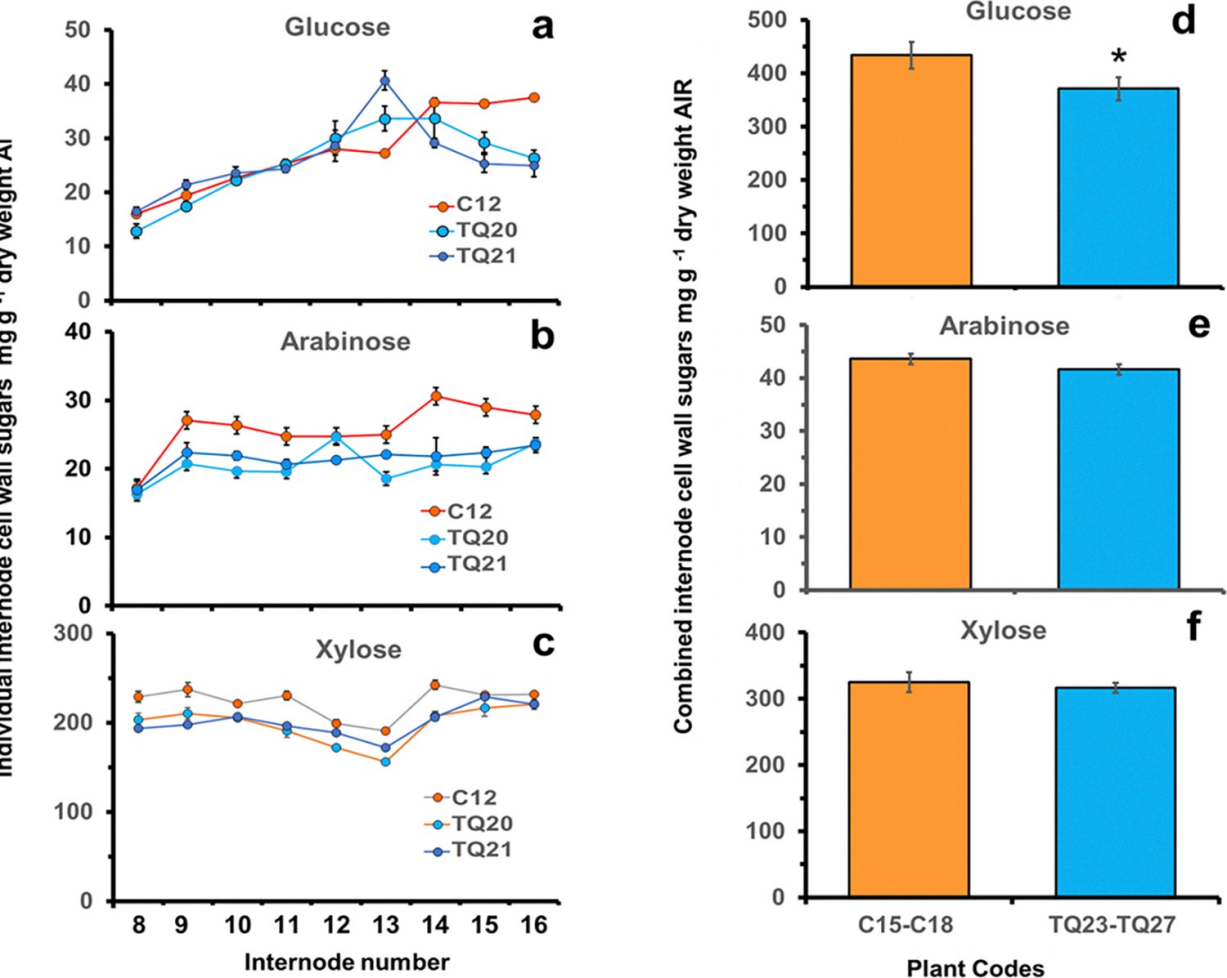
Mean levels of cell wall sugars of selected internodes of two FAEA expressing TQ and a control partner plant at the R+ stage of development **(a-c)** extracted by the trifluoracetic acid (TFA) method. Mean internode values ± sem (n = 3), and of combined internodes from single plants of 4 control and 5 TQ plants at the R stage of development **(d-f)** extracted with the TFA+sulphuric acid method. Bars are mean ± sem (C n = 12, TQ n = 15). Glucose **(a, d)** arabinose **(b, e)** xylose **(c, f).** * Indicates significant differences from controls (Student’s α = 0.05) (P<0.0471).

Cell walls were isolated as AIR from individual plant internodes and sugar analysis was conducted using TFA hydrolysis. As TFA does not hydrolyze crystalline cellulose, the glucose released by TFA treatment alone is likely from short β-1,4-glucan chains belonging to non-crystalline cellulose. Individual internodes of TQ20 and TQ21 (R+ stage), contained less arabinose and xylose, but similar levels of glucose (Fig 4A–4C). The overall xylose/arabinose ratio was lower in internodes of transgenic lines TQ20 and TQ21 compared with controls.

Sugar analysis was also conducted on the combined internodes of five additional TQ plants at the R stage of development, after both TFA and sulphuric acid (SA) hydrolysis. The levels of cell wall sugars showed some variation between TQ plants compared with controls (S7A Fig). Overall, the arabinose and xylose cell wall sugar contents were similar between TQ and control plants (Fig 4E and 4F) but glucose levels were statistically lower in TQ internodes compared with control internodes (Fig 4D). Plants TQ23, TQ27 and TQ24 contained less glucose, TQ23, TQ25, TQ27 less arabinose, but similar amounts of xylose compared with controls. In contrast, plants TQ26 and TQ25 contained similar levels of glucose and arabinose (except for TQ25 that contained less), and less levels of xylose (S7A–S7C Fig). The xylose/arabinose ratio was lower for three out of the five TQ plants compared with controls.

The relative proportion of arabinose, xylose and glucose released by the TFA alone or with the TFA + SA methods in the 4 controls and 5 TQ plants (Fig 4D–4F), is shown in S2A–S2C Fig. As expected, arabinose and xylose, which are polysaccharides H-bound to cellulose, were mainly removed by TFA hydrolysis, with values ranging from 90–94% of the cell wall arabinose, and from 80–88% of the cell wall xylose being recovered in the TFA fraction (S2A and S2B Fig). In agreement with the finding that TFA does not hydrolyse glycosidic linkages of crystalline cellulose, the release of glucose by TFA alone was from 29–35% of total cell wall cellulose for controls and from 8–35% for TQ internodes (S2C Fig). Most of the glucose was released following the SA hydrolysis, with amounts ranging from 71% for controls, and from 64–91% for TQ internodes (S2C Fig). It should be stressed that the total amount of glucose released by SA hydrolysis was similar between transgenic plants and controls, except for the pooled internodes from plant TQ24, which had a much lower level of released glucose (S2C Fig). However, when accounting for all the glucose released (combining SA and TFA), plants TQ23 and TQ27 showed lower levels compared with controls.

### Saccharification: Cellulase and xylanase-mediated glucose release from stover

The effect of inducible plant expressed FAEA on the recalcitrance of maize internode cell walls to enzymatic deconstruction was determined with internode AIR biomass samples from controls and TQ transgenic plants subjected to enzymatic saccharification. Initially this was done with *T*. *reesei* cellulase alone and then combined with *T*. *reesei* β-1,4 endo-xylanase and finally with Novozymes commercial CTec3 HS saccharification enzyme mixture. The released reducing sugars were determined using the PABAH method.

### Saccharification with *T*. *reesei* cellulase

**Fig 5:**
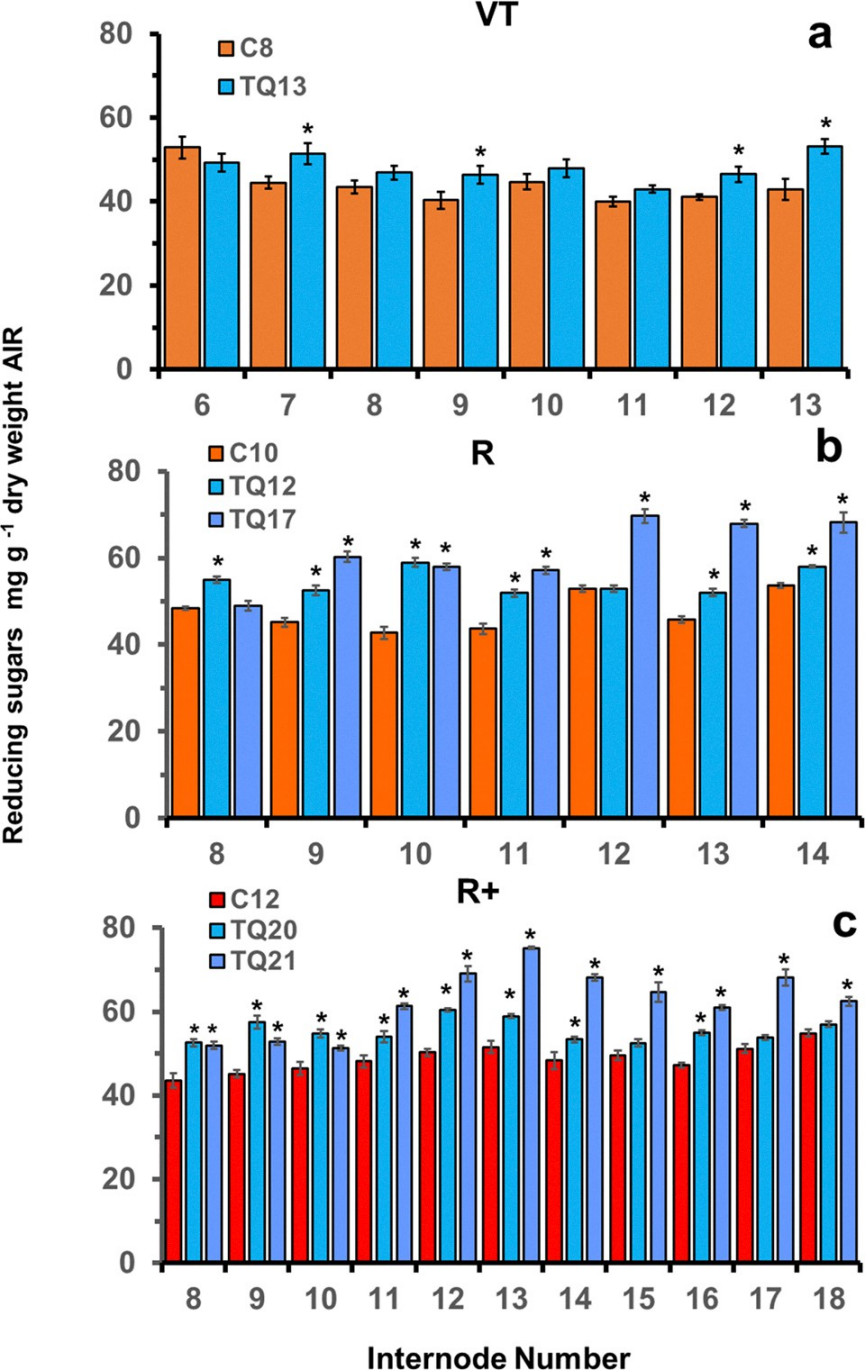
Mean levels of *T*. *reesei* cellulase mediated reducing sugars release from individual internodes of FAEA expressing and partner control plants at VT **(a)** R **(b)** and R+ **(c)** developmental stages. Freeze-dried buffer washed samples were treated with 336 U g^-1^ dry weight whole tissue of cellulase at 37°C for 48h, and the released sugars determined as total reducing sugars by the PABAH method using glucose as standard. Mean values ± sem (n = 4–8). * indicates significant differences from controls (Student’s α = 0.05).

Initially cell walls of individual internodes of FAEA expressing and partner control plants were treated with 336 U g^-1^ dry weight whole tissue of *T*. *reesei* cellulase at 37°C for 48 h and sugar release determined for 5 TQ and 3 control plants at three late developmental stages (VT, R and R+). Mean increases in sugar release in different internodes of FAEA-expressing plants above control plants were up to 24% for TQ13 (VT stage), 38% for TQ12 and 52% for TQ17 (R stage), and up to 27% for TQ 20 and 46% for TQ 21 (R+ stage) (Fig 5A–5C). The number of internodes showing significantly more sugar release than the corresponding control internodes also increased at the R+ stage as the plants entered late senescence (Fig 5B and 5C).

Different biomass to enzyme loadings of *T*. *reesei* cellulase, with AIR extracted from pooled whole plant internodes at the R stage, were used to optimize cellulase treatments and showed that sugar release from both the control plant C15 and plant TQ25 began to saturate at 2,500 U g ^−1^ dry weight AIR of cellulase (S4A Fig). Pairwise comparisons between TQ25 and C15 showed statistically significant increased levels of sugar released from TQ25 internodes over controls for cellulase loads between 500 (P< 0.0046) and 5,000 U g ^−1^ dry weight AIR (P< 0.001) (S4A Fig).

#### Saccharification with *T*. *reesei* cellulase plus *T*. *reesei* β-1,4 endo-xylanase

The combination of *T*. *reesei* cellulase (2,500 U g ^−1^ dry weight AIR) and different loadings of *T*. *reesei* β-1,4 endo-xylanase from 0 to 5,000 U g ^−1^ dry weight AIR did not have as high an impact on sugar release from internodes as expected. However, endo-xylanase addition did result in a statistically significantly higher sugar release from internodes of plant TQ25 compared with the control plant C15, for all xylanase loads tested (P<0.0001) (S4B Fig).

Sugar release by 2,500 U g ^−1^ dry weight AIR *T*. *reesei* cellulase over a 72-h period from pooled internodes of control plant C15 and two FAEA plants, began to saturate by 48h, at which point 75–80% of the total sugars of both control and TQ plants were released by the enzymes (S4D Fig), with TQ plants releasing significantly more sugars than the control plant at all time points. Equivalent results were observed when 2,500 U g ^−1^ dry weight AIR endo-xylanase was used in combination with 2,500 U g ^−1^ dry weight AIR *T*. *reesei* cellulase, but with higher amounts of sugar release than with cellulase alone (S4E Fig).

**Fig 6:**
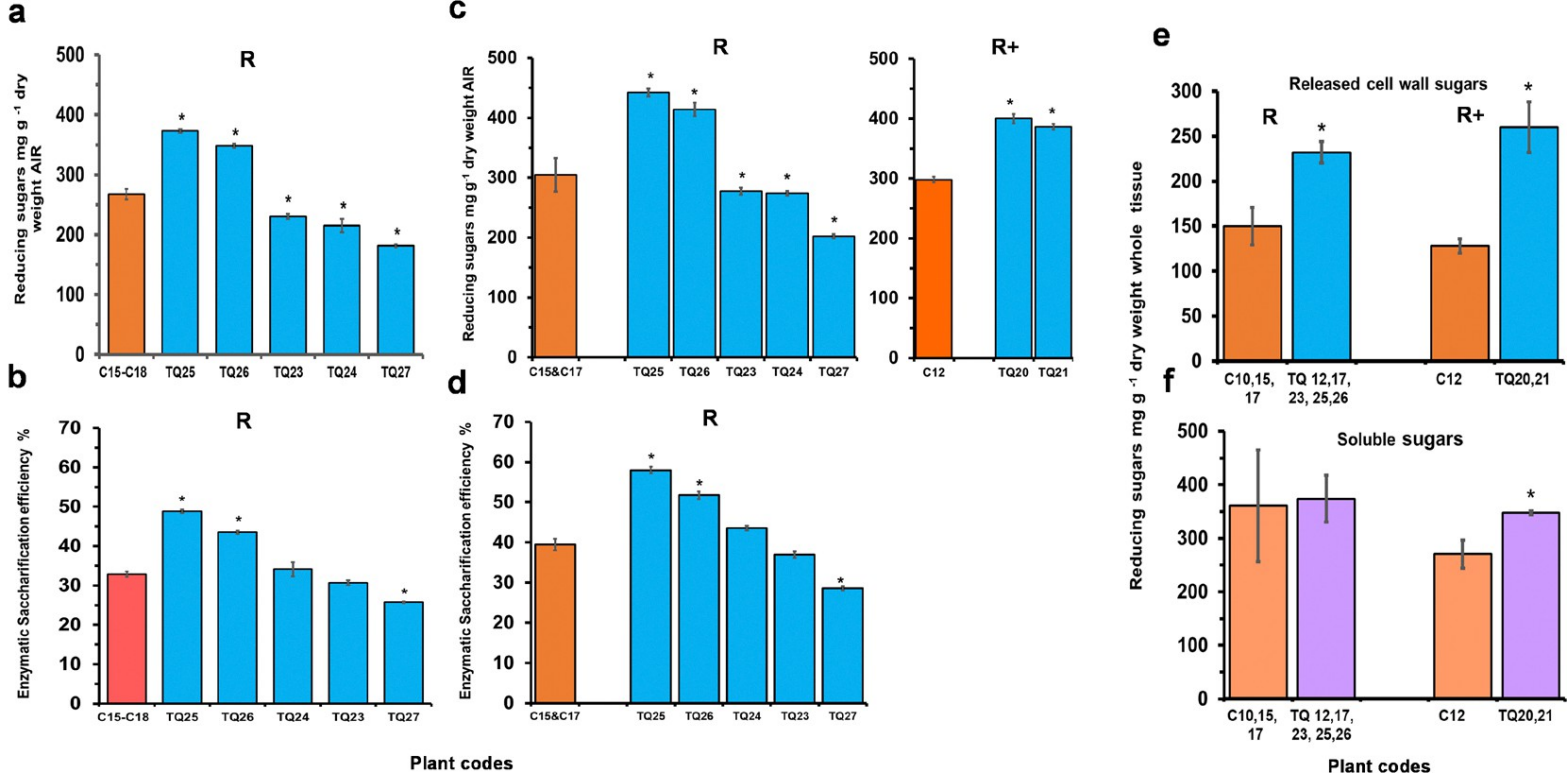
The effect of *T*. *resii* cellulase (5000 Units g^-1^AIR) + GCI140 xylanase (5000 Units g^-1^ dry weight AIR) on release of reducing sugars from pooled internode cell walls of 4 control (C15-C18) and 5 TQ plants (TQ23-TQ27) at the R stage **(a)** and saccharification efficiency **(b)**, with tissues treated at 37°C for 72 h, and the effect of CTec3 HS (250 Units g^-1^ dry weight AIR) on release of reducing sugars **(c)** and on saccharification efficiency **(d)** of 4 control (C12, C15-C17) and 7 TQ plants at the R (TQ23-TQ27) or R+ (TQ20, TQ21) stages of development, with tissues treated at 45°C for 72h. Mean values ± sem (n = 4–5). * Indicates significant differences from controls (Student’s α = 0.05), (P< 0.0001). Soluble and cell wall bound reducing sugars released by CTec3 HS (250 Units g^-1^ dry weight) from unextracted combined internodes from individual plants at the R and R+ stages of development. Released wall bound sugars **(e)**, soluble sugars **(f)**. Whole unextracted freeze dried tissues was incubated at 45°C for 72 h in the presence and absence of CTec3 HS enzyme. Mean values ± sem. (TQ n = 40–59, C n = 8–20). *Indicates significant differences from controls (Student’s α = 0.05), (P< 0.0001).

Treatment of pooled internode cell walls of 4 control and 5 TQ plants with a mixture of *T*. *reesei* cellulase and *T*. *reesei* β-1,4 endo-xylanase (5,000 U g^−1^), resulted in significant increases in the levels of soluble sugars released from plants TQ26 and TQ25, (P< 0.0001) (Fig 6A). Saccharification efficiency, measured as the total reducing sugars released as a percentage of total cell wall sugars in the tissue, significantly increased by up to 48% in TQ 25 and TQ 26 plants compared with the mean of the 4 control plants (Fig 6B).

### Saccharification with Cellic® CTec3 HS, (Novozymes)

The proprietary enzyme mixture in Cellic® CTec3 HS, (Novozymes North America Inc.), (a blend of cellulases, bacterial β-glucosidase and a range of hemicellulases) was also tested as the biocatalyst is one of the most efficient enzyme cocktails for lignocellulosic biomass hydrolysis. CTec3 HS was found to be more effective in cell wall sugar hydrolysis compared to *T*.*r*. cellulase or *T*.*r*. cellulase + GC140 xylanase digests. For example, while 50 U CTec3 HS g^−1^ dry weight AIR released 288 and 390 mg g−^1^ dry weight AIR reducing sugars from controls and TQ25 internodes respectively (S4C Fig), cellulase+xylanase required 2,500 U g^-1^ dry weight AIR of each enzyme in order to release equivalent amounts of sugars (S4B Fig).

Pairwise comparisons between TQ25 and C15 showed statistically significant increases in the level of sugars released from TQ25 internodes for all CTec3 HS loads tested (P< 0.001) (S4C Fig). Sugar release by 250 U g^-1^ dry weight AIR CTec3HS enzyme over a 72-h period from pooled internodes of control plant C15 and two FAEA plants, began to saturate by 48h, at which point 84–89% of the total sugars of both control and TQ plants were released by the enzymes (S4F Fig), with TQ plants releasing significantly more sugars than the control plant at all time points.

The treatment of pooled internode cell walls of two controls and five TQ plants at the R stage and two TQ plants and respective control at the R+ stage with 250 U g^-1^ dry weight AIR CTec3 HS resulted in a significant increase in the level of soluble sugars released from TQ plants at both the R and R+ stage from 33–43% compared with the corresponding control plants (Fig 6C). Saccharification efficiency for internodes of TQ plants also increased significantly in 2 of the 5 plants tested by up to 48% compared with controls (Fig 6D).

HPAEC analysis of solubilized cell wall sugars released by *T*. *reesei* cellulase + xylanase (GC140) (2,500 U g^-1^ dry weight AIR) or CTec3 HS (250 U g^-1^ dry weight AIR) confirmed the results where total reducing sugars was measured using the PABAH method (S5B Fig), with 23–30% of arabinose plus xylose contributing to total reducing sugars.

In order to assess the contribution of soluble internode sugars and the effect of using cell wall AIR, combined, but unextracted, internode tissues of 3 control and 5 TQ plants at the R stage and 2 TQ plants at the R+ stages, were treated with and without 250 U g^-1^ dry weight of the enzyme mixture CTec3 HS. While the soluble sugars varied between both controls and TQ plants (Fig 6F, S7C Fig), a significant increase in released wall bound sugars was found for all the FAEA expressing plants tested (Fig 6E, S7C Fig).

The percentage increase in sugar released from TQ internodes ranged from 29–78% in R stage plants and from 81–125% in R+ stage plants, compared with respective controls (Fig 6E, S7C Fig), higher than found with the use of AIR for these determinations (Fig 6C).

HPLC analysis of cellulase enzyme digests with cellulase, or a mixture of cellulase and xylanase or with Ctec3 HS also showed the presence of free ferulate monomers released from all the TQ plants analysed [S6Ia, S6Ib, S6IIc and S6IId Fig]. Cellulase enzyme digests of internode AIR from control plants also showed the release of several other feruloylated cell wall components, possibly feruloylated arabinoxylans [S6Ia, S6Ib, S6IIa, S6IIb, S6bIIIa, S6IIIc Fig]. The level of free ferulate monomers in TQ plants increased with the addition of xylanase [S6Ib, S6IIc, S6IId Fig], even though extracted AIR lacks any FAEA activity and was higher in three of the five TQ plants than in controls [S6I Fig (b1 insert)]. When enzyme digests from controls were then subjected to a mild TFA hydrolysis [S6IIIb, S6IIId Fig] or TFA hydrolysis followed by saponification [S6IVa, S6IVb Fig], both TQ and control digests showed the same free ferulate monomers.

Individual data set points behind the graphs presented in the figures (Figs 1–6 and S1–S7 Figs) can be found in S1 Table.

## Discussion

Corn stover constitutes one of the most sustainable lignocellulosic biomasses for bioconversion to ethanol [3]. However, ferulates present in grass cell walls and their ability to cross-link AX chains, or AX and lignin [11] constitute key factors that increase cell wall recalcitrance in grasses [16, 17]. Genetic manipulation of biomass cell wall structure/composition has been an important approach taken aiming to reduce biomass recalcitrance, however in many instances this approach results in negatively affecting plant fitness and total biomass, as shown by constitutive expression of FAEA or endo-xylanase in different grass species [32, 24, 25] and reflects the important roles that both ferulates and xylans play in grass cell walls. Overexpression of an endo-glucanase gene from the thermophilic bacterium *Acidothermus cellulolyticus* [44] or of the rice exo-glucanase gene (*EXG1*) [45] in the cytosol or apoplast in rice, also resulted in shorter stature as well as in developmental defects such as leaf blade separation, necrosis and sterility. However, when FAEA was placed under a *Lolium multiflorum* senescence enhanced promoter the TQ plants generated were not phenotypically different from the non-transformed control plants and showed normal development and biomass. Furthermore, this approach was clearly shown to be effective in altering the level of cell wall ferulates and cross-linking throughout the stover of mature maize plants [25].

In the present study we have made use of these TQ plants to investigate how FAEA expression changes with prolonged plant growth and during subsequent senescence, and the effect apoplastic FAEA activity has on cell wall ferulates, acetyl bromide lignin, and on stover digestion and saccharification. From previous study we have stablished that when under the inducible *Lm*See1 promoter, FAEA expression in maize internode expansion was not induced until after the fast elongation stage had passed [25]. As shown here, FAEA expression increased as plants reached the R stage and declined on prolonged plant senescence to the R+ stage, both in leaves and in internodes (Fig 1A–1C). The increase of FAEA activity in internodes as plants mature and senesce, is consistent with further reductions in the levels of cell wall ester-linked ferulate monomers and dimers [8–5’-DFA; 5–5’-DFA; 8-O-4’-DFA; 8–5’-DFA (benzofuran cyclic form) and an unknown FD], in internodes of TQ plants at the R and R+ stages of development (Fig 2A and 2B). The broader range of ferulate dimers released with FAEA expression *in planta* is again in agreement with our previous findings [23, 22, 46] and in contrast with results from *in vitro* studies which have shown that *A*. *niger* FAEA can only hydrolyse ferulates monomers ester-linked to arabinose and 5–5’DFA and 8-O-4’DFA in small amounts, from Driselase-treated cell walls [47] or when in combination with xylanase [48]. Together, these results suggest that when expressed *in planta*, FAEA have higher accessibility to the cell wall esterified ferulates. The removal of ferulate monomers and dimers by FAEA expression *in planta*, may also be preventing and/or reducing ferulic acid cross-linking between AX chains and between AX and lignin. An interesting finding was the increase in *p*-coumaric levels in internodes of TQ plants at the R and R+ developmental stages. Ferulates anchored to the cell wall have the dual ability to cross-link AX chains to one another (via ester linkages) in addition to cross-link AX-lignin, via ester-ether linkages, strengthening the cell wall and increasing its recalcitrance [5, 9, 11, 15]. Changes in the level of ferulates in the cell wall as a result of FAEA expression, might be triggering compensation mechanisms in TQ plants. Although there is no evidence that *p*-Coumaric acid is involved in AX-lignin cross-link, these phenolics acylates lignin monomers, assisting with lignin polymerization [49], thus compensating for the reduced levels of ferulates which are important participant in the lignification process by anchoring lignin to the wall.

Further evidence from mild acid hydrolysis also shows that the level of ferulates ester-linked to AX and to lignin (the residue fraction) have been significantly reduced in TQ plants (S2D Fig). It has been reported that some plant species have evolved to produce monolignol (ML) ferulate conjugates that can be incorporated into lignin. This is a process that involves ferulates being coupled to CoA (catalysed by CoA ligase) and then to monolignols to form ML-FA conjugates (catalysed by feruloyl-CoA: monolignol transferase) [50, 51]. Originally, we hypothesized that ferulates being removed from the wall could potentially be used by these enzymes to generate ML-FA. However, preliminary data from TFA-mild acid hydrolysis followed by saponification of cell wall residues from combined internodes from two TQ plants compared with control internodes, showed no increase in ferulic acid ester-linked to lignin in the cell wall residue (S2D Fig). In fact, FA incorporated into lignin will not be released by saponification. This is not the case for *p*CA as it does not readily couple.

Interestingly, FAEA activity in internodes of TQ plants harvested at the R stage of development, was relatively stable, with no loss in activity during the first 8 weeks when stored at 4°C, but with up to 18% loss when stored at room temperature (Fig 1G). The stability of any *in planta* expressed enzymes is quite an important factor when considering their application in industrial processes utilising post-harvest feedstocks. Although FAEA activity is not likely to increase during post-harvest if the tissue is no longer metabolically active, the accumulated FAEA in the intact tissue may continue to act on the ester-linked cell wall ferulates, if the water content is high enough, and if time is given under the right conditions.

While it may be generally considered that the acetyl bromide method gives a good estimate of the total lignin content of tissues [40, 43, 52], the interpretation of the AB lignin results reported here are extremely difficult to explain if we assume this to be the case.

We suspect that the increase in lignin content of AIR from TQ plants may only be apparent as we previously found a significant increase of AB lignin in *Festuca* leaves co-expressing apoplast-targeted FAEA and apoplast-targeted ß-1,4-endo-xylanase (XYN2) but with no increase in Klason lignin [15].

This apparent increase in lignin content of AIR may be due either to an increase in HCAs, and in particular *p*CA from TQ cell walls, which show higher levels of *p*CA (Fig 2A and S7B Fig), increasing the absorption at 280nm when released by AB solubilisation, and/or an increased loss of cell wall carbohydrates from TQ cell walls due to the action of FAEA reducing cross-links to arabinoxylans and to lignin, following the more severe extraction method used in AB determinations, resulting in a subsequent increase in lignin density in extracted cell walls of TQ internodes compared with control plants.

Morrison and Stewart (1995) [53] raised the issues related to the presence of significant levels of cell wall-bound HCAs in sample material, and as these compounds also absorb at 280nm, the same wavelength used in lignin quantification, they could potentially contribute to the total lignin determination. Brinkmann et al. (2002) [52] also addressed the issues with HCAs in grasses and reported that the “lignin” concentration determined with the AB method was strongly dependent on whether or not the cell walls were subjected to alkaline hydrolysis to remove covalently bound aromatic non-ligneous components before” lignin determination”. These findings suggest that what we may be seeing is that FAEA expression has resulted in an increase in the extractability with acetyl bromide of non-ligneous components with absorption at 280 nM. Dean [39] addresses the presence of vast arrays of phenolic compounds present in plants as a factor that can interferes with lignin quantification protocols and states that the removal of these compounds is likely to lead to removal of some of the lignin in samples but if not removed can contribute to the total lignin determination. However, as previously well addressed [49, 54, 55], as around half of the total ferulates and a great part of *p*-coumaric acid are linked to lignin, these should be regarded as part of the lignin complex in monocot cell walls.

Although treatment of freeze-dried, buffer washed, individual internodes with a low concentration of cellulase alone resulted in low levels of soluble reducing sugars released, significantly higher levels of soluble sugars were released, from the TQ internodes than from the controls at all stages of development with sugar release increasing particularly during late-stage senescence (Fig 5A–5C), and with increased cellulase concentration (S4A Fig). When cellulase was used in combination with xylanase with R stage plants, a significantly higher sugar release (Fig 6A, S4B Fig), as well as a higher saccharification efficiency was found for two transgenic lines (TQ25 and TQ26) which contained similar levels of total cell wall sugars as the control internodes (Fig 6B). Interestingly, these two transgenic lines also showed a 10–12% reduction in the level of cell wall xylose (S7A Fig). Quantification of the reducing sugars present in the enzymatic digest of pooled internodes by HPAEC showed that they contained xylose and arabinose as well as glucose, with a significantly higher release of glucose from both transgenic lines compared with controls (S5B Fig).

When Cellic® CTec3 HS replaced *T*.*r*. cellulase or *T*.*r*. cellulase + GC140 xylanase, it was found that only a fiftieth of CTec3 HS was required to release the same amount of sugar from AIR samples, and 4 out of 7 of the pooled internodes from TQ plants showed significantly higher levels of reducing sugars released compared with controls (Fig 6C).

However, some plants (e.g. TQ23 and TQ27), did not show a significantly increased sugar release from AIR, above control levels when treated with either *T*. *resii* cellulase + GCI140 xylanase or with CTec3 HS (Fig 6). This may be due to lower FAEA activity in these plants (Fig 1A and 1D), or a failure in some segregating T_1_ progeny to maintain apoplastic targeting of FAEA, resulting in relatively high FAE enzyme activity in the tissues, but with a lower effect on cell wall HCAs during plant development.

Interestingly, when unextracted combined internodes at R and R+ stages of development which contained high levels of FAEA enzyme activity, were treated with CTec3 HS, the levels of released cell wall bound sugars were significantly higher in all seven TQ lines tested compared with controls (Fig 6C; S7C Fig).

This increase in sugar yields from whole unextracted internode tissue when treated with CTec3 HS, compared with AIR, can be explained by a combination of two effects, firstly the direct effect of FAEA activity on the levels of cell wall HCAs during development (an effect that is present in both AIR and in unextracted tissue), and the high FAEA enzyme activity in unextracted tissues, but not in AIR, which contributes synergically with xylanase and cellulase [22] in the CTec3 HS enzyme mixture to enhance sugar release from the cell walls.

These results suggest that cellulose and hemicellulose are more easily hydrolysed in FAEA expressing plant internodes and might indicate that the reduction of ferulates and ferulates-AX cross-linking might account for an increased accessibility of enzymes to their respective substrates.

In addition, the finding that FAEA activity is quite stable during post-harvest with only up to 40% loss after 12 weeks when stored at room temperature (Fig 1G) indicates that the accumulated FAEA in the intact tissue may continue to act on the ester-linked cell wall ferulates, during saccharification reducing the levels of CW ferulates even further and contributing to the higher level of sugar release in all the transgenic lines tested compared to controls when unextracted internodes were treated with CTec3 HS under the saccharification conditions.

An interesting and intriguing finding involved the presence of only free ferulate monomers in enzyme digests from TQ plants, but bound forms in control digests, whether using cellulase alone, cellulase + xylanase or the enzyme cocktail in CTec3 HS. It is unlikely that these bound HCAs are enzyme contaminants, or they would have also appeared in TQ plant digests.

We propose a major mechanism responsible for the enhanced saccharification in transgenic FAEA expressing plants as the result of the direct effect of ester-linked ferulate monomers and dimers being removed from the wall by FAEA, thus exposing the hemicellulose to hydrolysis by xylanases. In addition, the removal of ferulate monomers by FAEA is likely to result in less ferulates available for oxidative dimerization and consequently leading to reduced AX cross-linking, thereby facilitating AX hydrolysis by xylanases. The negative impact of cell wall bound ferulates on saccharification, as indicated in this study, is consistent with previous reports where a significant reduction in cell wall bound ferulates, as a result of the overexpression of a BAHD acyltransferase (*Os*AT10) in rice [56] and switchgrass [57], or the downregulation of SvBADH01 in *Setaria viridis* [58] which resulted in significantly enhanced saccharification efficiency.

## Conclusion

Biomass recalcitrance, which hinders the conversion of cell wall polysaccharides to sugars, requires pre-treatment and enzyme cocktail technology, rendering the process economically challenging. We suggest that a fungal FAEA under a senescence promoter, can be activated *in planta*, at later developmental stages and throughout the stover of mature maize plants, without compromising plant fitness and/or total biomass, and contribute to the improvement of biomass saccharification efficiency by making sugars more extractable with hydrolytic enzymes.

Evidence suggests that the mechanism for increase in saccharification efficiency is at least in part, the result of the direct effect of ester-linked ferulate monomers and dimers being removed from the wall by FAEA, exposing the hemicellulose to hydrolysis by arabinofuranosidases and xylanases. The removal of ester-linked ferulates by FAEA, by leaving arabinose more prone to be recycled from the wall, could in turn affect the ability of the xylan backbone to adsorb onto cellulose, thus potentially facilitating the hydrolysis of cellulose and hemicellulose during the saccharification process.

Future research is needed to test these plants for bioethanol production under novel process conditions. For example, these plants may not need complex pre-treatment or the addition of FAE during fermentation for the conversion of the lignocellulose material to biofuels. It also raises the realistic possibility of expressing other wall degrading enzymes such as xylanase and cellulase under such a senescence promoter to generate plants that self-saccharify at senescence. To further understand the potential of FAEA, it will be also important to assess its compatibility with C5 and C6 co-fermenting microorganisms for the efficient processing of xylose together with cellulose, so that both polysaccharides can contribute to the carbohydrate pool for fermentation to ethanol. Furthermore, it will be interesting to study the effect of FAEA expression on AX recycling and the impact of xylan adsorption onto cellulose, to further understand plant expressed FAEA effects on enhanced saccharification. In addition, it will be important to establish if plants from different transformation events, which are homozygous for FAEA, significantly differ in terms of enzyme activities and self-digestion, from heterozygous plants. Since arabinoxylan feruloylation has a significant impact on cell wall structure and degradability, and its manipulation at later stages during plant development does not negatively impact plant development and biomass, our approach has the potential to improve biomass conversion to biofuels, digestibility as feed, as well as biomaterials from lignocellulose.

## Supporting information

S1 Fig Maximum internode FAEA activity from V6 to VR+ developmental stages in TQ plants with insert showing mean % dry weight of stover at each stage **(a).** Internode number with maximum FAEA activity **(b)** and Maximum leaf FAEA activity on a dry weight basis **(c).** V6—VT are means ± sem (n = 3) from single plants. R and R+ are the means of two plants ± SD (n = 6).
(TIF)

S2 Fig Levels of cell wall sugars extracted with the trifluoracetic acid (TFA) followed by the sulphuric acid (SA) methods. Arabinose **(a)** xylose **(b)** and glucose **(c)** of combined internodes of 4 control (C) and 5 TQ plants at the R stage of development. Mean ± sem (C n = 12, TQ n = 3). * Indicates significant differences from controls (Student’s α = 0.05) (P<0.0049–0.075). Wall bound HCAs released by mild hydrolysis with trifluroacetic acid (TFA) and saponification of TFA-fraction and pellet from combined internodes of plants C17 and TQ23, TQ25. Ferulic acid and *p*-coumaric acid ester-linked to arabinoxylan (supernatant) **(d)** and to the lignin (pellet) **(e)**. * Indicates significant differences from controls (Student’s α = 0.05) (P< 0.0143–0.0049).
(TIF)

S3 Fig Levels of ester and ether linked HCAs [*p*-coumaric acid (*p*CA), ferulate monomers (FM) and ferulate dimers (FD)] in internodes 9–12 in control plants C8 and C5 **(a, c)** and in plants TQ13 and TQ10 **(b, d)** at the VT stage of development. * Indicates significant differences from controls (Student’s α = 0.05). (P<0.039–0.0001).
(TIF)

S4 Fig Effect of *T. reesei* cellulase **(a, d)**, *T. reesei* cellulase + GC140xylanase **(b, e)** and CTec3 HS enzyme **(c, f)** loading **(a, b, c)** and kinetics **(d, e, f)** on reducing sugar release from combined internodes of control C and TQ plants at the R stage of development. For cellulase and cellulase + xylanase enzyme loading **(a-b)** samples were incubated at 37°C for 72 h and for CTec3 HS, enzyme loading **(c)** at 45°C for 72 h. For the kinetics of sugar release **(d-f)** samples were treated with cellulase (2500 Units g-1 dry weight AIR) **(d)** or cellulase + xylanase (2500 Units g-1 dry weight AIR) **(e)** or CTec3 HS (250 Units g-1 dry weight AIR) **(f)**. Mean values ± sem (n = 3). * Indicates significant differences from controls (Student’s α = 0.05) (P< 0.0046–0.0001).
(TIF)

S5 Fig Comparison of different loadings of CTec3 HS and *T*. *reesei* cellulase on the release of reducing sugars from combined internodes of control C15 and TQ25 plants. AIR samples were incubated for 72 h in 50mM Na acetate buffer pH 5 or in 100mM Na acetate buffer pH 5.5 in the presence of CTec3 HS (45°C) or cellulase (37°C) enzymes **(a)**. HPAEC analysis of solubilised cell wall sugars released by *T*. *resii* cellulase *(*Millipore) + xylanase (GC140) (2500 Units g^-1^ dry weight AIR) or CTec3 HS (Novozymes Cellic) (250 Units g^-1^ dry weight AIR^)^ **(b).** AIR extracted from internodes of control and TQ plants were treated with either *T*. *resii* cellulase + GC140 xylanase for 72 h at 37°C or with CTec3 for 72 h at 45°C, and reducing sugars determine by the PABAH method. * Indicates significant differences from controls (Student’s α = 0.05) (P< 0.0249–0.0001) [a] and (P< 0.015–0.0001) [b].
(TIF)

S6 Fig HPLC chromatograms and UV spectra of ferulates released from pooled internodes of four control [C15, C16, C18 and C20) and four FAEA expressing plants [TQ17, TQ23,TQ25 and TQ29), by the action of *T*. *reesei* cellulase on plants C15 & TQ25 (I a) or a mixture of cellulase and xylanase on plants C15 & TQ23 **(I b)** and the amounts of free ferulate released **(I b1)**, and from plants C16 **(II a)**, C17 **(II b)**, TQ24 **(II c)** and TQ25 **(II d)** and the effects of TFA hydrolysis **(III c, d)** on feruloylated compounds released by the action of cellulase **(III a)** or Ctec3 HS **(III b)** from control plants C18 and C20. Also, the effects of TFA hydrolysis and NaOH saponification on ferulates released by the action of cellulase on pooled internodes of pants C15 **(IV a)**, C20 **(IV b)**, TQ25 **(IV c)** and TQ29 (**IV d)**. The peak at 6.6 min = Internal standard 2-hydroxycinnamic acid. For **(I), (II)** and **(IV)** AIR was extracted from internodes and treated with 5000 Units g ^-1^ *T*. *resii* cellulase or 5000 Units g ^-1^ cellulase + 5000 Units g ^-1^ GCI140 xylanase for 72 h at 37°C. For **(III)** AIR was extracted from internodes and treated with 5000 Units g ^-1^ *T*. *resii* cellulase or 250 Units g ^-1^ or Ctec3 HS for 72 h at 37°C and the supernatants were then hydrolysed with 50 mM TFA for four hours at 99°C **(III c & III d)**, and then saponified with 2M NaOH at RT for 16 hr **(IV a-d)**.
(TIF)

S7 Fig **a.** Mean levels of cell wall glucose, arabinose and xylose of combined internodes of four control (C15-C18) and five TQ plants (TQ23-TQ27) at the R stage of development extracted with the TFA+sulphuric acid method (**a**). Bars are mean ± sem (C n = 12, TQ n = 3).). * Indicates significant differences from controls (Student’s α = 0.05). **b.** Levels of ester-linked HCAs in the cell walls of combined internodes of four control (C15 -C18) and five TQ plants (TQ23-TQ27) at the R stage of development **(b).** *p*-coumaric acid (*p*CA), ferulate monomers (FM) and total ferulate dimers (FD). Error bars are mean values ± sem (n = 3). * Indicates significant differences from controls (Student’s α = 0.05) (P< 0.0633–0.0001-). **c.** Soluble and cell wall bound reducing sugars released by CTec3 HS enzyme from unextracted combined internodes at the R and R+ stages of development of three control (C10, C15 & C17) and five TQ plants (TQ12, TQ17, TQ23. TQ25 & TQ26) at the R stage, and plants C12 and TQ20 and TQ21 at the R+ stage **(c)**. Whole unextracted freeze dried powdered internode tissues were incubated at 45°C for 72 h in 50mM Na acetate buffer pH 5 in the presence and absence of 250 Units g^−1^ dry weight CTec3 HS enzyme.
(TIF)

S1 Table Individual data set points behind the graphs presented in the figures (Figs 1–6 and S1–S7 Figs). (XLSX)
