# Supplementary figures and images for: Late-stage changes in the composition of cell walls of maize plants expressing an apoplast targeted, senescence enhanced fungal ferulic acid esterase, and the subsequent effects on tissue saccharification
Source: PLoS One. 2025 Jan 3;20(1):e0315950. doi: 10.1371/journal.pone.0315950 (PMC11698336; doi:10.1371/journal.pone.0315950)

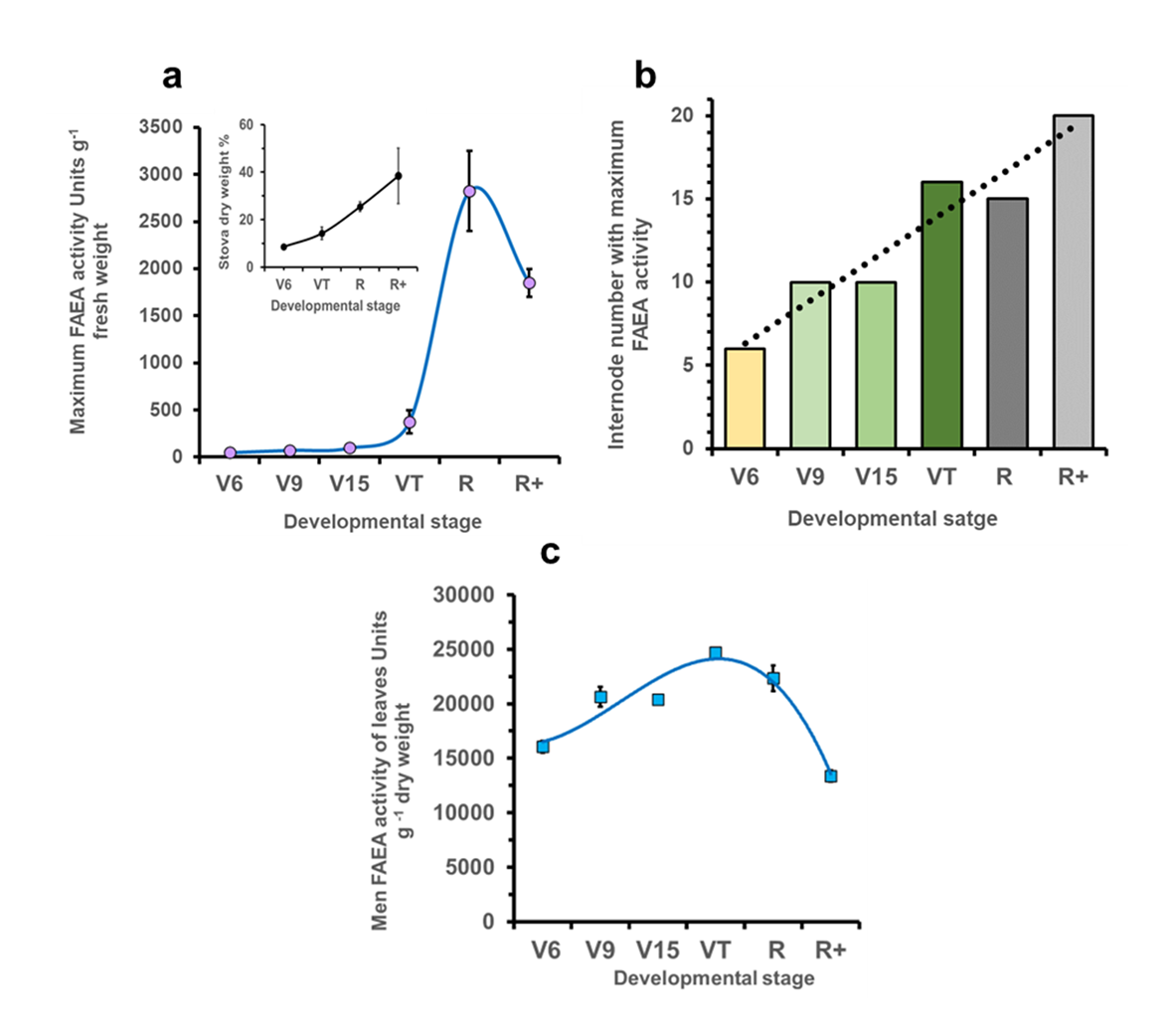

Supplement: S1 Fig — Maximum internode FAEA activity from V6 to VR+ developmental stages in TQ plants with insert showing mean % dry weight of stover at each stage (a). Internode number with maximum FAEA activity (b) and Maximum leaf FAEA activity on a dry weight basis (c). V6—VT are means ± sem (n = 3) from single plants. R and R+ are the means of two plants ± SD (n = 6). (TIF) [file pone.0315950.s001.tif]

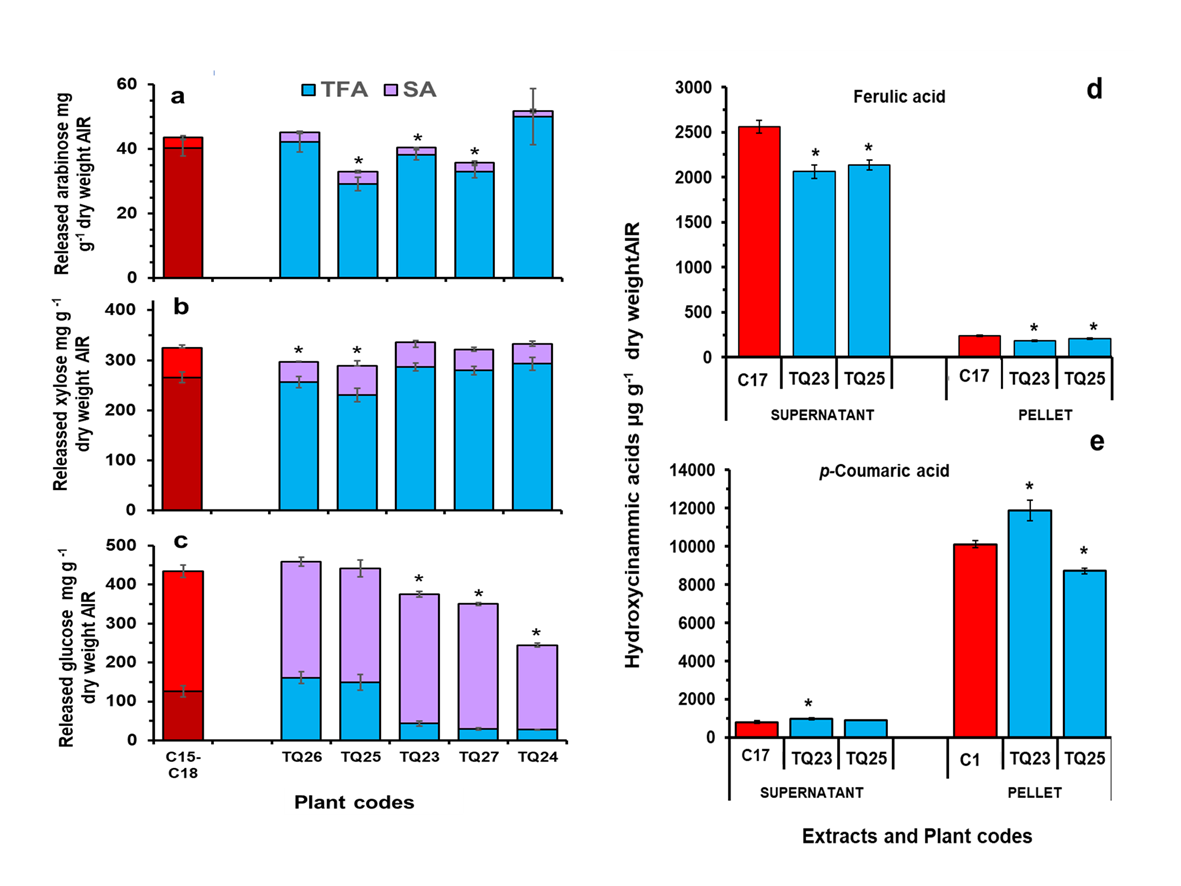

Supplement: S2 Fig — Arabinose (a) xylose (b) and glucose (c) of combined internodes of 4 control (C) and 5 TQ plants at the R stage of development. Mean ± sem (C n = 12, TQ n = 3). * Indicates significant differences from controls (Student’s α = 0.05) (P<0.0049–0.075). Wall bound HCAs released by mild hydrolysis with trifluroacetic acid (TFA) and saponification of TFA-fraction and pellet from combined internodes of plants C17 and TQ23, TQ25. Ferulic acid and p-coumaric acid ester-linked to arabinoxylan (supernatant) (d) and to the lignin (pellet) (e). * Indicates significant differences from controls (Student’s α = 0.05) (P< 0.0143–0.0049). (TIF) [file pone.0315950.s002.tif]

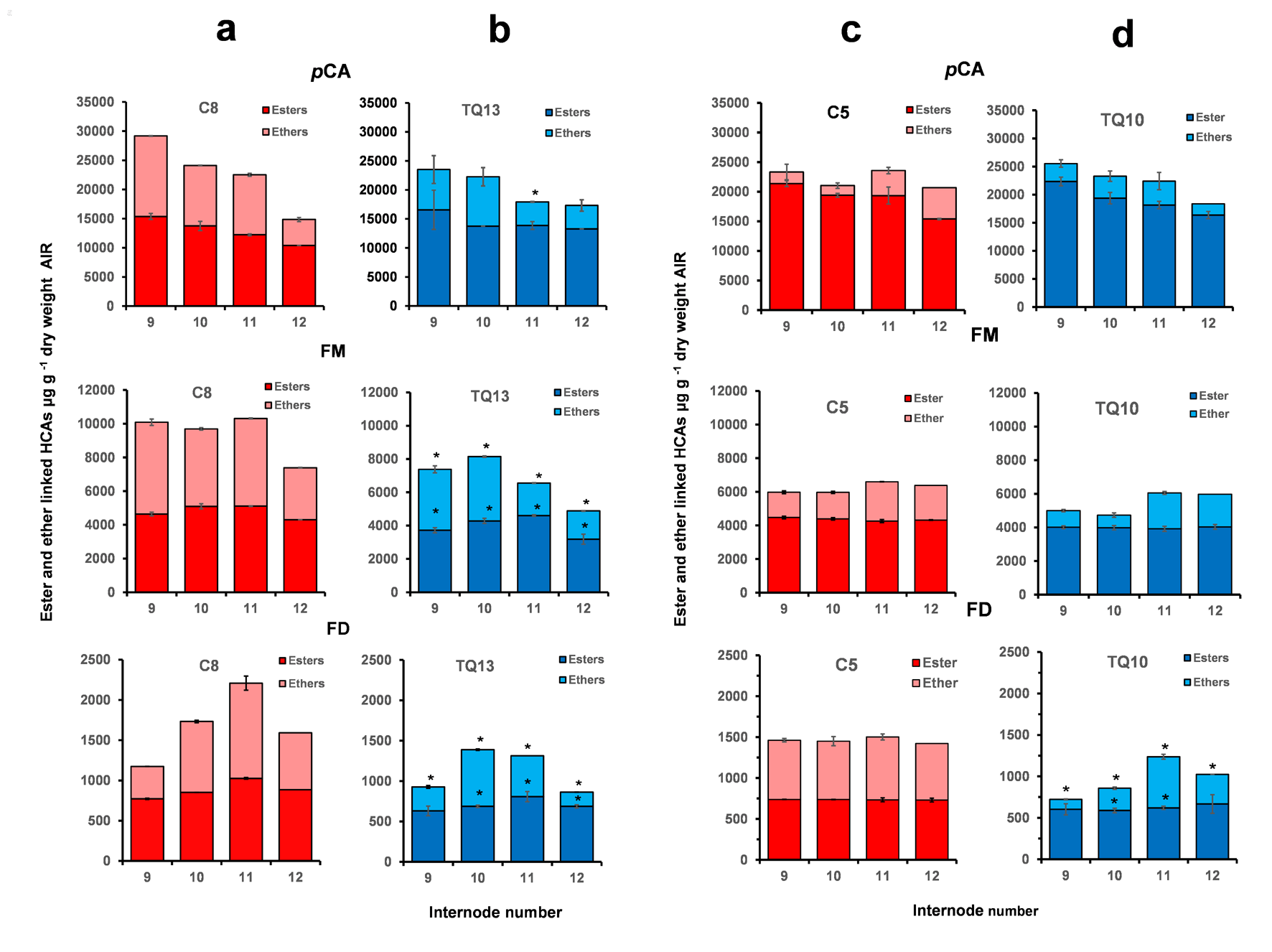

Supplement: S3 Fig — Levels of ester and ether linked HCAs [p-coumaric acid (pCA), ferulate monomers (FM) and ferulate dimers (FD)] in internodes 9–12 in control plants C8 and C5 (a, c) and in plants TQ13 and TQ10 (b, d) at the VT stage of development. * Indicates significant differences from controls (Student’s α = 0.05). (P<0.039–0.0001). (TIF) [file pone.0315950.s003.tif]

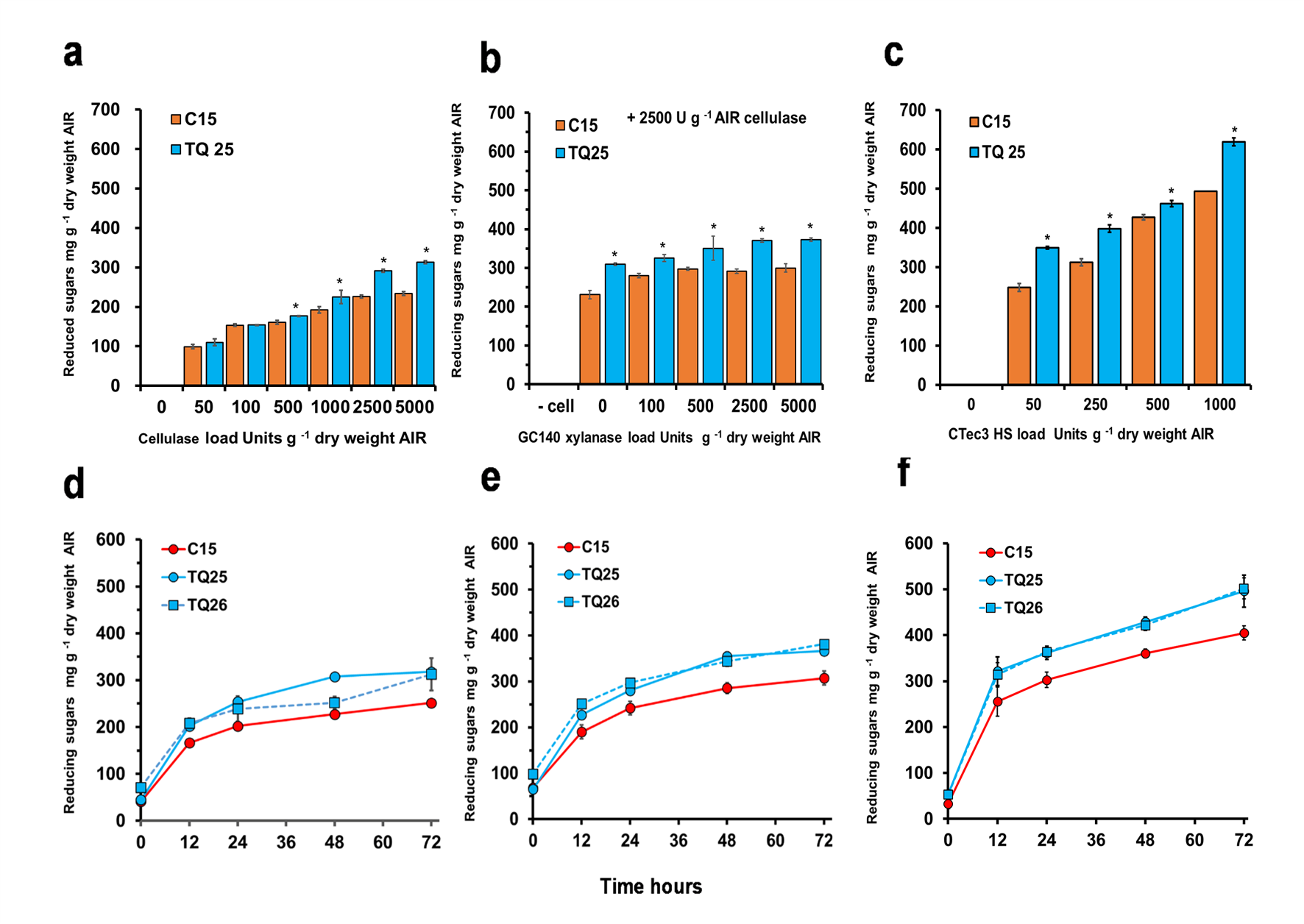

Supplement: S4 Fig — Effect of T. reesei cellulase (a, d), T. reesei cellulase + GC140xylanase (b, e) and CTec3 HS enzyme (c, f) loading (a, b, c) and kinetics (d, e, f) on reducing sugar release from combined internodes of control C and TQ plants at the R stage of development. For cellulase and cellulase + xylanase enzyme loading (a-b) samples were incubated at 37°C for 72 h and for CTec3 HS, enzyme loading (c) at 45°C for 72 h. For the kinetics of sugar release (d-f) samples were treated with cellulase (2500 Units g-1 dry weight AIR) (d) or cellulase + xylanase (2500 Units g-1 dry weight AIR) (e) or CTec3 HS (250 Units g-1 dry weight AIR) (f). Mean values ± sem (n = 3). * Indicates significant differences from controls (Student’s α = 0.05) (P< 0.0046–0.0001). (TIF) [file pone.0315950.s004.tif]

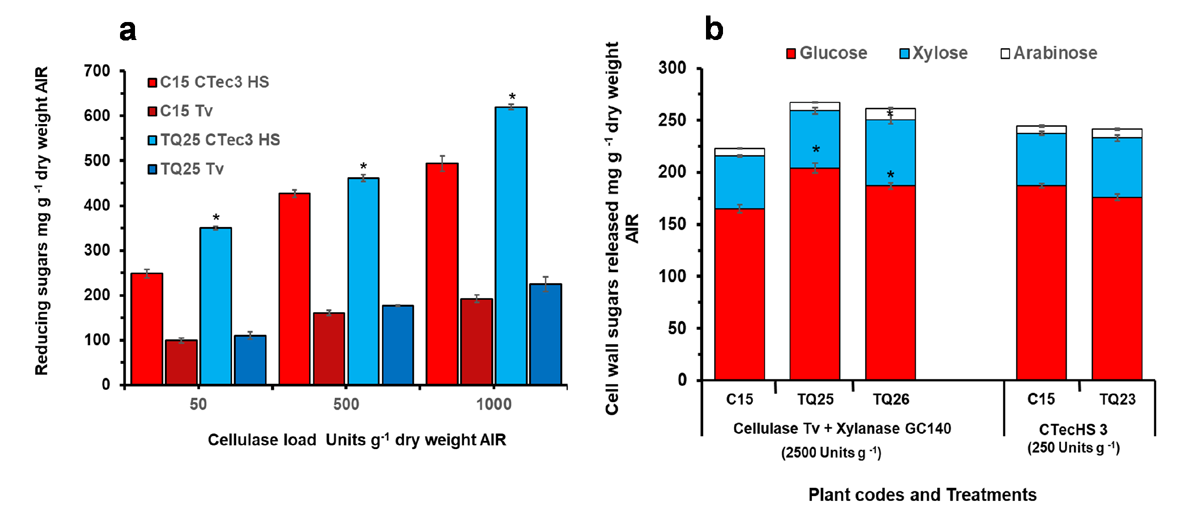

Supplement: S5 Fig — AIR samples were incubated for 72 h in 50mM Na acetate buffer pH 5 or in 100mM Na acetate buffer pH 5.5 in the presence of CTec3 HS (45°C) or cellulase (37°C) enzymes (a). HPAEC analysis of solubilised cell wall sugars released by T. resii cellulase (Millipore) + xylanase (GC140) (2500 Units g-1 dry weight AIR) or CTec3 HS (Novozymes Cellic) (250 Units g-1 dry weight AIR) (b). AIR extracted from internodes of control and TQ plants were treated with either T. resii cellulase + GC140 xylanase for 72 h at 37°C or with CTec3 for 72 h at 45°C, and reducing sugars determine by the PABAH method. * Indicates significant differences from controls (Student’s α = 0.05) (P< 0.0249–0.0001) [a] and (P< 0.015–0.0001) [b]. (TIF) [file pone.0315950.s005.tif]

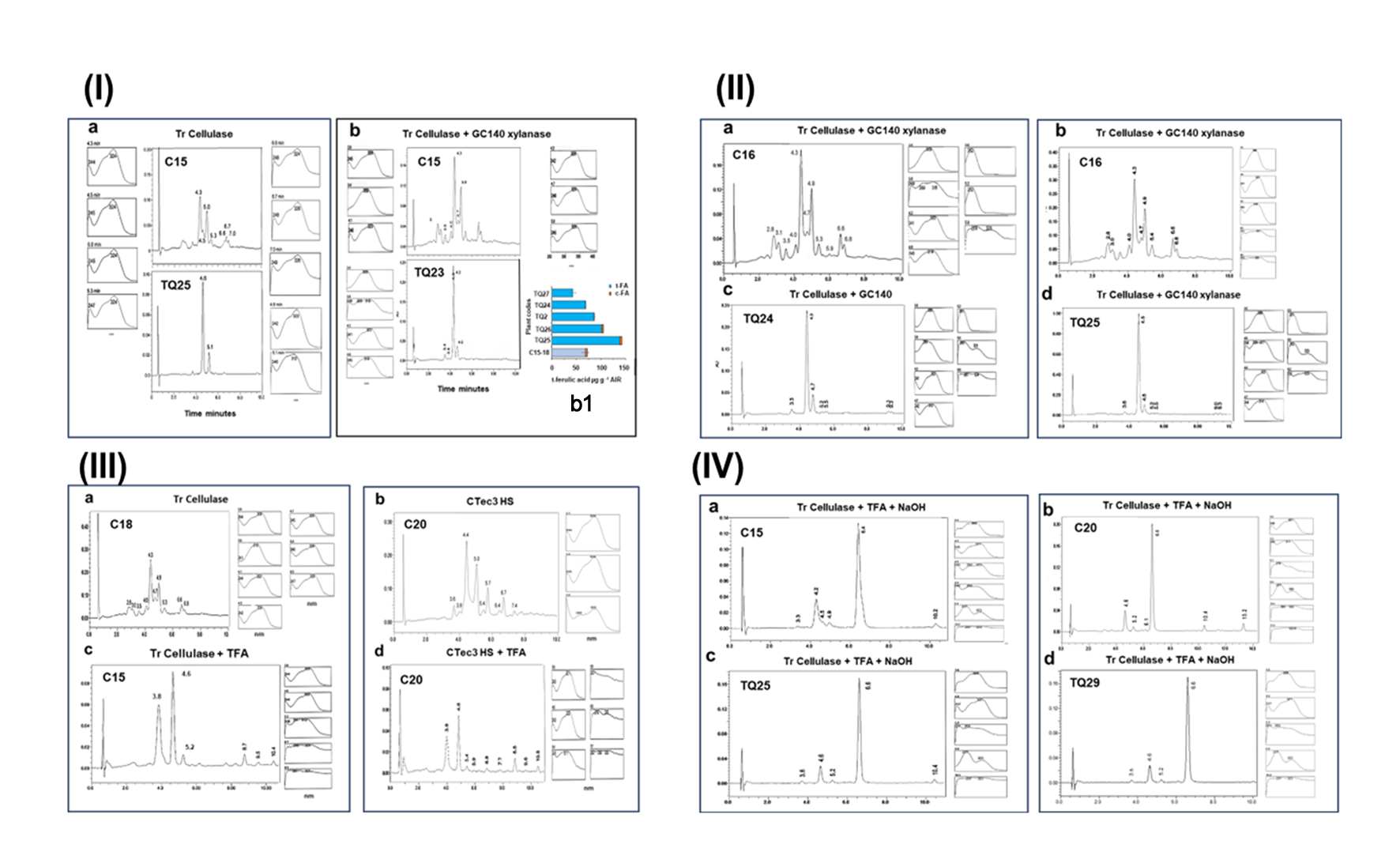

Supplement: S6 Fig — HPLC chromatograms and UV spectra of ferulates released from pooled internodes of four control [C15, C16, C18 and C20) and four FAEA expressing plants [TQ17, TQ23,TQ25 and TQ29), by the action of T. reesei cellulase on plants C15 & TQ25 (I a) or a mixture of cellulase and xylanase on plants C15 & TQ23 (I b) and the amounts of free ferulate released (I b1), and from plants C16 (II a), C17 (II b), TQ24 (II c) and TQ25 (II d) and the effects of TFA hydrolysis (III c, d) on feruloylated compounds released by the action of cellulase (III a) or Ctec3 HS (III b) from control plants C18 and C20. Also, the effects of TFA hydrolysis and NaOH saponification on ferulates released by the action of cellulase on pooled internodes of pants C15 (IV a), C20 (IV b), TQ25 (IV c) and TQ29 (IV d). The peak at 6.6 min = Internal standard 2-hydroxycinnamic acid. For (I), (II) and (IV) AIR was extracted from internodes and treated with 5000 Units g -1 T. resii cellulase or 5000 Units g -1 cellulase + 5000 Units g -1 GCI140 xylanase for 72 h at 37°C. For (III) AIR was extracted from internodes and treated with 5000 Units g -1 T. resii cellulase or 250 Units g -1 or Ctec3 HS for 72 h at 37°C and the supernatants were then hydrolysed with 50 mM TFA for four hours at 99°C (III c & III d), and then saponified with 2M NaOH at RT for 16 hr (IV a-d). (TIF) [file pone.0315950.s006.tif]

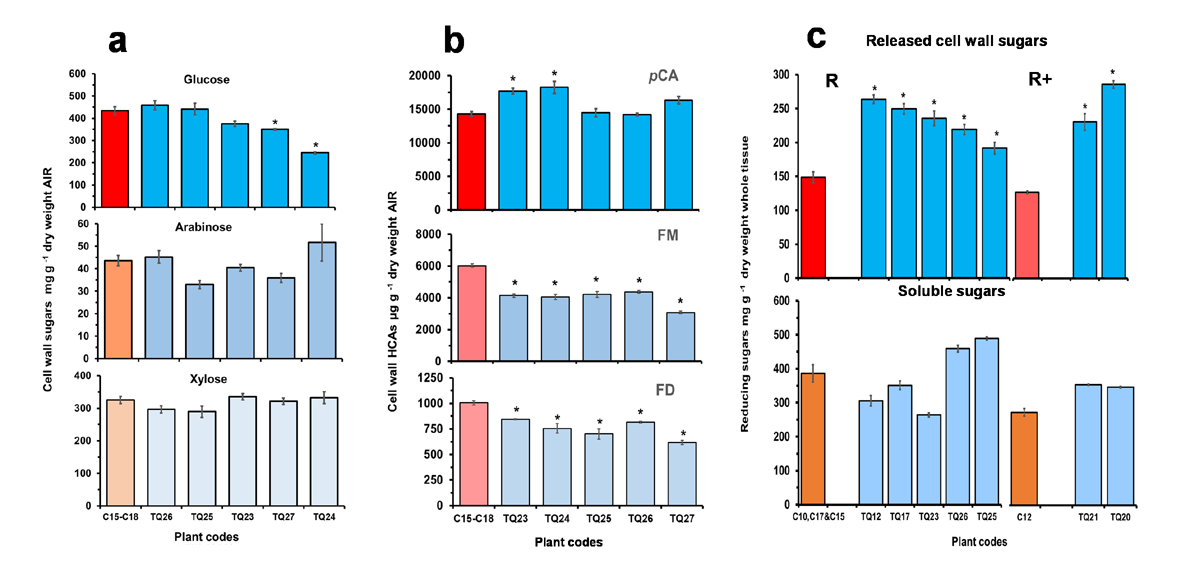

Supplement: S7 Fig — a. Mean levels of cell wall glucose, arabinose and xylose of combined internodes of four control (C15-C18) and five TQ plants (TQ23-TQ27) at the R stage of development extracted with the TFA+sulphuric acid method (a). Bars are mean ± sem (C n = 12, TQ n = 3).). * Indicates significant differences from controls (Student’s α = 0.05). b. Levels of ester-linked HCAs in the cell walls of combined internodes of four control (C15 -C18) and five TQ plants (TQ23-TQ27) at the R stage of development (b). p-coumaric acid (pCA), ferulate monomers (FM) and total ferulate dimers (FD). Error bars are mean values ± sem (n = 3). * Indicates significant differences from controls (Student’s α = 0.05) (P< 0.0633–0.0001-). c. Soluble and cell wall bound reducing sugars released by CTec3 HS enzyme from unextracted combined internodes at the R and R+ stages of development of three control (C10, C15 & C17) and five TQ plants (TQ12, TQ17, TQ23. TQ25 & TQ26) at the R stage, and plants C12 and TQ20 and TQ21 at the R+ stage (c). Whole unextracted freeze dried powdered internode tissues were incubated at 45°C for 72 h in 50mM Na acetate buffer pH 5 in the presence and absence of 250 Units g−1 dry weight CTec3 HS enzyme. (TIF) [file pone.0315950.s007.tif]
